# Rewiring of the protein–protein–metabolite interactome during the diauxic shift in yeast

**DOI:** 10.1007/s00018-022-04569-8

**Published:** 2022-10-15

**Authors:** Dennis Schlossarek, Marcin Luzarowski, Ewelina M. Sokołowska, Venkatesh P. Thirumalaikumar, Lisa Dengler, Lothar Willmitzer, Jennifer C. Ewald, Aleksandra Skirycz

**Affiliations:** 1 Department of Molecular Physiology, Max Planck Institute of Molecular Plant Physiology, 14476 Potsdam, Germany; 2 Core Facility for Mass Spectrometry and Proteomics, ZMBH, Universität Heidelberg, 69120 Heidelberg, Germany; 3 Boyce Thompson Institute, Cornell University, Ithaca, 14850 USA; 4 Interfaculty Institute of Cell Biology, Eberhard Karls University of Tuebingen, Tuebingen, Germany

**Keywords:** Yeast, Diauxic-shift, Co-fractionantion mass spectrometry, Protein-metabolite interactions, Dipeptides, Proteasome

## Abstract

In budding yeast *Saccharomyces cerevisiae*, the switch from aerobic fermentation to respiratory growth is separated by a period of growth arrest, known as the diauxic shift, accompanied by a significant metabolic rewiring, including the derepression of gluconeogenesis and the establishment of mitochondrial respiration. Previous studies reported hundreds of proteins and tens of metabolites accumulating differentially across the diauxic shift transition. To assess the differences in the protein–protein (PPIs) and protein–metabolite interactions (PMIs) yeast samples harvested in the glucose-utilizing, fermentative phase, ethanol-utilizing and early stationary respiratory phases were analysed using isothermal shift assay (iTSA) and a co-fractionation mass spectrometry approach, PROMIS. Whereas iTSA monitors changes in protein stability and is informative towards protein interaction status, PROMIS uses co-elution to delineate putative PPIs and PMIs. The resulting dataset comprises 1627 proteins and 247 metabolites, hundreds of proteins and tens of metabolites characterized by differential thermal stability and/or fractionation profile, constituting a novel resource to be mined for the regulatory PPIs and PMIs. The examples discussed here include (i) dissociation of the core and regulatory particle of the proteasome in the early stationary phase, (ii) the differential binding of a co-factor pyridoxal phosphate to the enzymes of amino acid metabolism and (iii) the putative, phase-specific interactions between proline-containing dipeptides and enzymes of central carbon metabolism.

**Supplementary Information:**

The online version contains supplementary material available at 10.1007/s00018-022-04569-8.

## Introduction

Budding yeast S*accharomyces cerevisiae* grown on glucose undergoes two growth phases: glucose-utilizing and ethanol-utilizing phases, separated by a period of growth arrest, referred to as diauxic shift [75]. The depletion of both glucose and ethanol is associated with the cell cycle arrest and the onset of the stationary phase [70]. During the glucose-utilizing phase, yeast uses glucose to produce ATP and pyruvate through glycolysis. Pyruvate is further converted to ethanol, which accumulates in the medium until glucose is depleted. The presence of glucose suppresses oxidative phosphorylation (OXPHOS), gluconeogenesis and the use of alternative carbon sources. The repression of respiration in the presence of oxygen is referred to as the Crabtree effect and is reminiscent of the Warburg effect described for cancer cells [27]. Decreasing glucose concentration triggers first gradual and, once glucose is depleted, abrupt changes to the metabolism characterized by de-repression of gluconeogenesis and establishment of mitochondrial respiration [75].

The transition from glucose to ethanol-based growth is orchestrated by the central energy signalling pathways, including the PKA (cAMP-dependent protein kinase), TORC1 (target of rapamycin) and Snf1 (sucrose non-fermenting) kinases [10, 22]. Downstream targets of PKA, TORC1 and Snf1 comprise multiple transcriptional regulators, which drive the massive transcriptional reprogramming reported for the diauxic shift [20]. Changes in gene expression are reflected at the protein and metabolite levels, hundreds of proteins and tens of metabolites displaying differential accumulation across glucose to ethanol transition [44]. For instance, abundance of glycolytic enzymes and metabolic intermediates decreases, whereas enzymes and metabolites of the glyoxylate cycle accumulate during the diauxic shift [75].

Biological entities, such as proteins and metabolites, rarely act independently but rather as a part of a larger complex. Resulting protein–protein (PP) and protein–metabolite (PM) interactions have diverse functional consequences from structural to regulatory with implications to all known cellular processes. The dramatic changes in the protein and metabolite abundance reported for the diauxic shift [44, 75] likely translate into a significant rewiring of the PP and PM interactome. New interactions can be driven by the changes in the protein and metabolite concentrations and an alteration in the interactor status, such as, for instance, by a post-translational modification (PTM) of the protein partner. However, although the importance of protein interactions is undisputed, many interactions are still poorly characterized. This is especially true for protein–metabolite interactions.

While there are many approaches to study PPIs and PMIs, only a few allow a bird-eye view into the entirety of the interactomes across cellular transitions, such as the diauxic shift. One such method is thermal proteome profiling (TPP) [54]. Here, the interaction status of a protein is gauged from the difference in the temperature stability expressed as melting temperature—the temperature at which 50% of a protein is unfolded, compared between the different conditions [42]. Initially developed to look for protein partners of small-molecule ligands [54], TPP has been successfully applied to assess global changes in the protein interactomes [5, 19]. The change in the temperature stability measured between two cellular states, whether it is a genetic, environmental or developmental perturbation [5, 19, 39] is indicative of a change in the protein interaction status, where the interacting partner can be, e.g. a protein, metabolite or nucleic acid. Protein thermal stability can be also affected by the change in the PTMs status [30, 49, 59]. TPP experiments are ideal to assess the global change to a protein interactome. Moreover, as proteins in a complex show coordinated changes in their melting behaviour when done across a large number of cell states, TPP experiments can be used to predict the composition of protein complexes [39].

A complementary approach that can be used to capture differential interactomes is co-fractionation mass spectrometry (CF-MS) (reviewed, e.g. by Salas et al. [52]. CF-MS combines separation of complexes utilizing different biochemical techniques, such as size exclusion chromatography (SEC) [2, 29, 68], ion exchange (IEX) chromatography [14, 29], blue native gels [26] or density gradient centrifugation [24] with mass spectrometry (MS) analysis of the obtained fractions and uses co-elution to delineate interactors. First established for PPIs [14, 24, 26, 29, 40, 68], CF-MS methods can also be used to resolve protein–metabolite [14, 34, 36, 65] and protein–RNA interactions [37].

In the past we used a CF-MS approach, that we dubbed PROMIS for PRotein Metabolite Interactions using Size Separation to build a protein–metabolite interaction map of a budding yeast *Saccharomyces cerevisiae* during the glucose-utilizing, logarithmic growth stage [36]. In doing so we reported hundreds of known and unknown small molecules separating together with proteins, attesting to the previously postulated [33, 35] complexity of the protein–metabolite interactome, and the notion that many more small molecules than are known today interact with, and modulate the function of, their protein partners.

In the current study, we extended our analysis to explore the dynamics of PPIs and PPMs during the diauxic shift transition to understand how the dramatic changes in the protein and metabolite abundance reported for the diauxic shift would translate into a rewiring of the PP and PM interactome. To this end, yeast harvested in the glucose-utilizing, fermentative phase, ethanol-utilizing and early stationary respiratory phases were subjected to isothermal shift assay (iTSA, a variation on the TPP [4] and PROMIS analysis. We could demonstrate that the diauxic shift transition is associated with major changes in the PP and PM complexes. Moreover, our work attests to the suitability of PROMIS to capture changes in PMIs, as shown before for PP complexes [28].

## Results and discussion

### Proteomics analysis of the diauxic shift

**Fig. 1:**
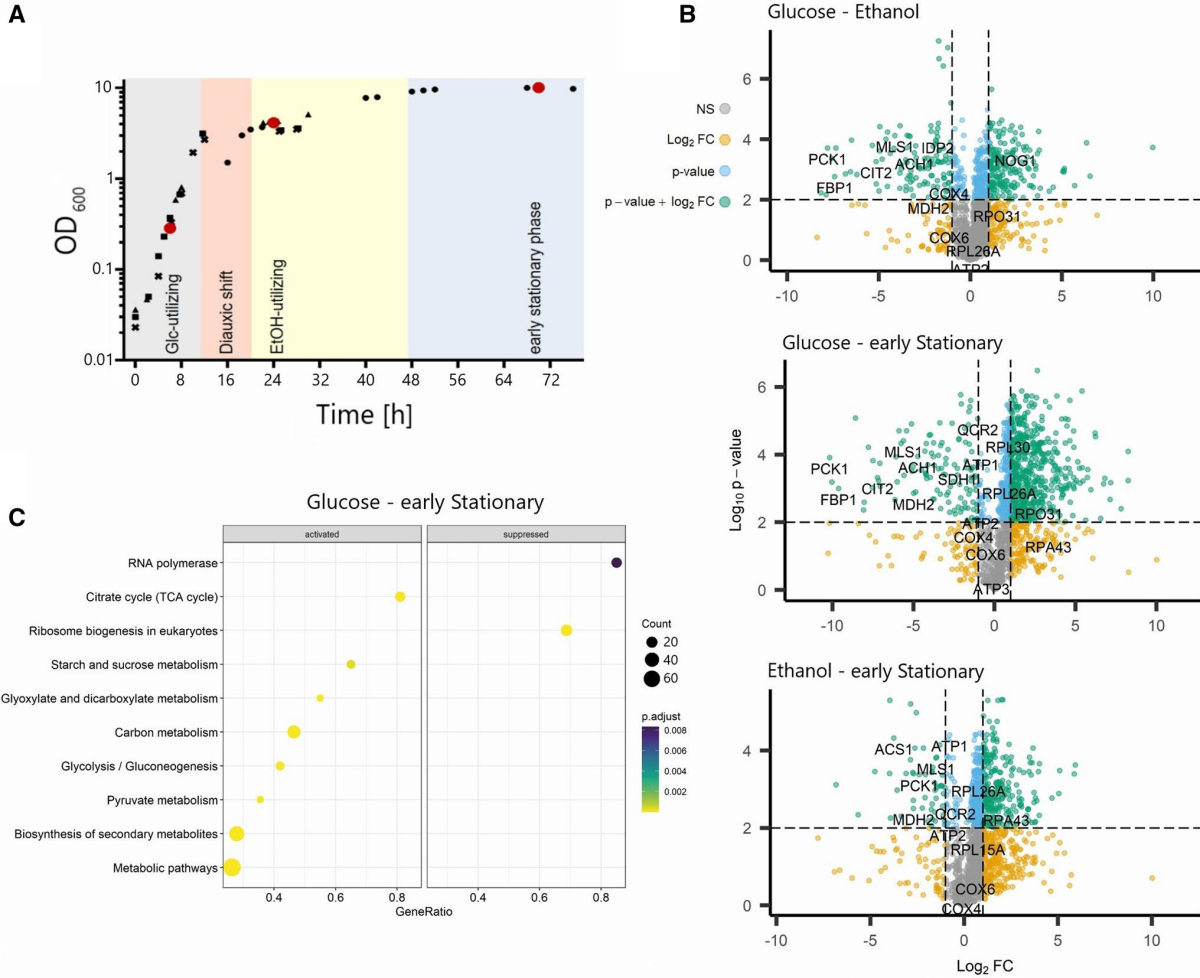
Changes in relative protein abundance between glucose- and ethanol-utilizing growth in *Saccharomyces cerevisiae*. **A** *S. cerevisiae* strain YSBN2 growth curve in glucose containing complete medium. Samples were collected after 6, 24 and 72 h corresponding to the glucose ethanol, and early stationary phases, respectively. **B** Volcano plot visualization of changes in relative protein abundances between growth phases tested using a two-sided *t*-test. The horizontal, dashed line indicates FDR = 0.01; vertical, dashed lines indicate a fold change greater than 2. Proteins with significant (FDR < 0.01) fold changes of more than 2 are highlighted in green, and proteins involved in central carbon metabolism are labelled. **C** KEGG enrichment analysis of proteins which significantly increase (activated) or decrease (suppressed) in abundance in the early stationary phase compared to the glucose-utilizing phase

To examine changes in the PM and PP interactome before and after the diauxic shift transition, we used the YSBN2 strain of *Saccharomyces cerevisiae*. YSBN2 is a strain closely related to the common model strain S288c and was constructed by Canelas et al. [13] for large-scale physiological studies. In contrast to S288c, YSBN2 is prototrophic, making it well suited for metabolic studies. It also carries a drug resistance marker to prevent contamination in large-scale or long-term cultures and to facilitate genetic crosses. OD600 was used to monitor growth and samples were taken after 6, 24 and 72 h of cultivation. The 6 h time point corresponds to the logarithmic, glucose-utilizing phase, the 24 h time point to post-diauxic, ethanol-utilizing phase and the 72 h time point to the early stationary phase (Fig. 1A and Fig. S1).

To examine how well our study compares with other diauxic shift omics and multi-omics experiments, we first measured protein abundances across the three sampled growth phases. Of the 1627 proteins present in our dataset, 697 proteins showed a significant (FDR < 0.01) fold change (FC) greater than 2 between the glucose-utilizing phase and early stationary phase, 300 between glucose-utilizing and ethanol-utilizing phase and 288 between ethanol-utilizing and early stationary phases, respectively (Fig. 1B, Tables S1–S2). The differential abundance of hundreds of proteins across yeast growth was reported before. For instance, authors of a comprehensive time-course proteomics study of the diauxic shift covering ten time points representing the switch from glucose to ethanol-based growth reported a differential abundance of approximately 50% of all measured proteins [44]. We next performed a KEGG enrichment analysis on the 697 proteins differing between glucose and early stationary phases (Fig. 1C). Among the 116 proteins we found accumulating in the early stationary in comparison to glucose-utilizing phase, proteins associated with the TCA cycle and glyoxylate and dicarboxylate metabolism were significantly enriched. The list included the rate-limiting enzymes of gluconeogenesis, fructose-1,6-bisphophatase (FBP1) and phosphoenolpyruvate carboxykinase (PCK1), TCA/glyoxylate cycle enzymes, such as citrate synthase (CIT1 and CIT2), and trehalose phosphate synthases (TPS1 and TPS2). This is in line with previous proteomics studies showing that the major metabolic events associated with the diauxic shift are reflected by the changes in enzyme accumulation and include the reversal of flux from glycolysis to gluconeogenesis, accumulation of trehalose and glycogen and activation of the TCA / glyoxylate cycle [44, 75]. Conversely, in accordance with what was reported by Murphy et al. [44], proteins associated with ribosome biogenesis and RNA polymerases were enriched among 581 downregulated proteins depleted in the early stationary versus glucose-utilizing phase. The list included the cytoplasmic GTPase RIA1 involved in the 60S ribosomal subunit biogenesis, ribosomal proteins (RPL29, RPS2), chaperones and RNA polymerase subunits, such as RPB3, RPB11 or TFG1.

In summary, proteomics analysis of the three sampled yeast growth phases revealed known signatures of diauxic shift transition.

### Thermal proteome profiling reveals major changes between fermentative and respiratory phase

**Fig. 2:**
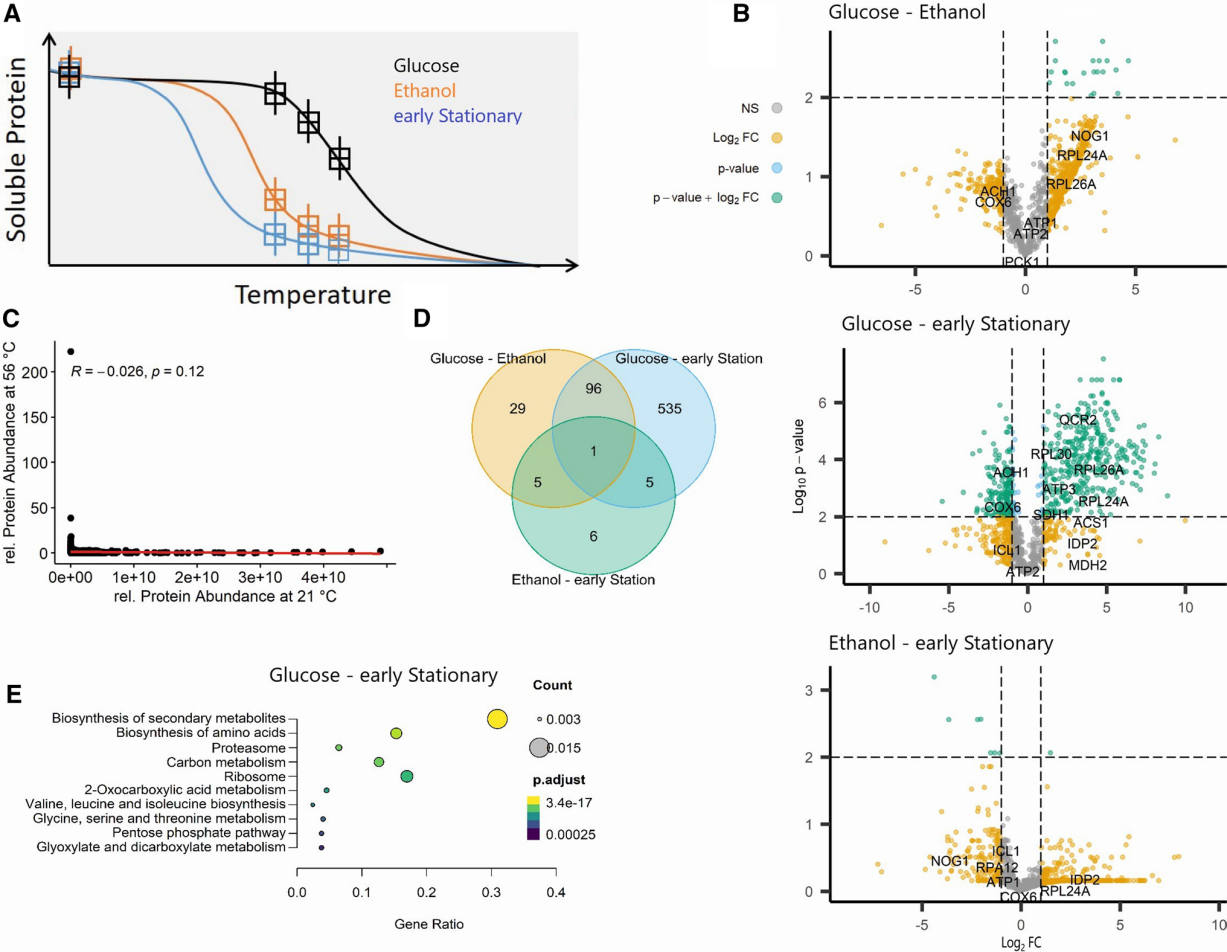
Growth phase-dependent changes in protein thermal stability. **A** Schematic differential thermal gradient curves of a protein sampled in three different growth phases. Protein thermal stability was measured after treatment with three elevated temperatures—48 °C, 52 °C and 56 °C—as described in materials and methods. **B** Volcano plots showing the differences in protein thermal stability at 56 °C between glucose- and ethanol-utilizing phase (upper panel), glucose- and early stationary phase (middle panel) and ethanol and early stationary phase (lower panel), respectively, tested using a two-sided *t*-test. The horizontal, dashed line indicates FDR = 0.01, vertical, dashed lines indicate a fold change greater than 2. Proteins with significant (FDR < 0.01) fold changes greater than 2 are highlighted in green, and proteins involved in central carbon metabolism are labelled. **C** Relative protein abundance and protein thermal stability at 56 °C are not correlated (PCC = -0.26). **D** Venn diagram showing the overlap of proteins with significant changes (FDR < 0.01, FC > 2) in thermal stability between the growth phases in all temperature treatments. **E** KEGG enrichment of the 97 proteins with significant changes in thermal stability between glucose-utilizing and early stationary phase. Only the ten most significant enrichments are shown

We next wanted to understand whether these major changes in protein abundance across the diauxic shift are accompanied by global changes in the protein interactome. We used a simplified thermal proteome profiling protocol called isothermal shift assay (iTSA) [4]. Instead of a temperature gradient, protein stability is measured in a single temperature selected based on the average melting temperature of the proteome [31] (Fig. 2A).

To better represent the “meltome” we used three different “melting temperatures”, 48, 52 and 56 °C. We additionally took samples at room temperature (RT) to normalize for the differences in the initial protein abundance. Statistical analysis revealed hundreds of differential proteins, the differences being most pronounced at 56 °C (Fig. S2). Interestingly, and again similarly to what was already reported [5, 66], there was no correlation between protein abundance at room temperature and thermal stability at 56 °C (Pearson correlation coefficient: − 0.026, *p* = 0.12) (Fig. 2C). Additionally, we could confirm a weak, but statistically significant negative correlation between a protein’s chain length and thermal stability, as described before [31, 32, 38] varying from *R* = − 0.064 in the early ethanol-utilizing phase to *R* = − 0.24 in the glucose-utilizing phase (Fig. S3).

We found that hundreds of proteins changed their stability across the three growth phases. Most differences (FDR < 0.05 and FC > 2 or < 0.5 at 48, 52 or 56 °C) were measured between the glucose and early stationary phases (Fig. 2B and D, Table S3), with fewer differences measured between consecutive growth phases. 637 proteins (53.5%) were significantly affected in their thermal stability between the glucose and early stationary, in comparison to 131 proteins (11% of all measured proteins) between the glucose and ethanol, and only 17 (1.4%) between the ethanol and early stationary phases. As would be expected 97 of the 131 proteins (74%) that differed between the glucose and ethanol also differed between the glucose and early stationary phases. A single protein, heat shock protein 26 (HSP26), was differential in all comparisons. Previously, using TPP Becher and colleagues reported that approximately 30% of all measured proteins displayed cell cycle-dependent changes in the melting stability [5]. While the two studies cannot be directly compared, they both demonstrate that cell state transitions are accompanied by significant changes in the thermal stability of hundreds of proteins.

A KEGG enrichment analysis on the 637 proteins changed between the glucose and early stationary phases (Fig. 2E) identified pathways involved in the biosynthesis of secondary metabolites (sce01110) and amino acids (sce01230), specifically valine, leucine and isoleucine biosynthesis (sce00290) and glycine, serine and threonine metabolism (sce00260), proteasome (sce03050), ribosome (sce03010) and carbon metabolism (sce01200). Further functional analysis using STRING database identified the presence of multiple subunits of seven macromolecular complexes: ribosome, proteasome, RNA polymerase complex, aminoacyl-tRNA synthetase complex, cytochrome c reductase complex, coatomer, CTT chaperone complex and V-ATPase (Fig. S3).

The reported differences in the thermal stability likely reflect changes either in a protein interaction or its PTM status [5, 30, 49, 56], or both, since PTMs are known to affect formation of PP and PM complexes [21]. Moreover, thermal stability can also serve as a proxy for enzyme activity, reflecting a change in the substrate occupancy [5]. In summary, proteome-wide analysis of changes in thermal stability across the transition from fermentative to respiratory metabolism reveals hundreds of proteins changing in stability, attesting to the significant changes in the protein interaction and/or PTMs status.

### The diauxic shift is associated with major changes in the metabolite–protein interactome

Building on the results from iTSA, to further examine growth phase-dependent changes in the protein–protein (PP) and protein–metabolite (PM) complexes, we used PROMIS [36, 57, 64, 65]. PROMIS relies on the size-based separation of molecular complexes present in native cell lysate: metabolites bound to protein complexes separate into earlier-eluting high molecular weight fractions, whereas unbound small molecules separate in late-eluting low molecular weight fractions [64, 65].

**Fig. 3:**
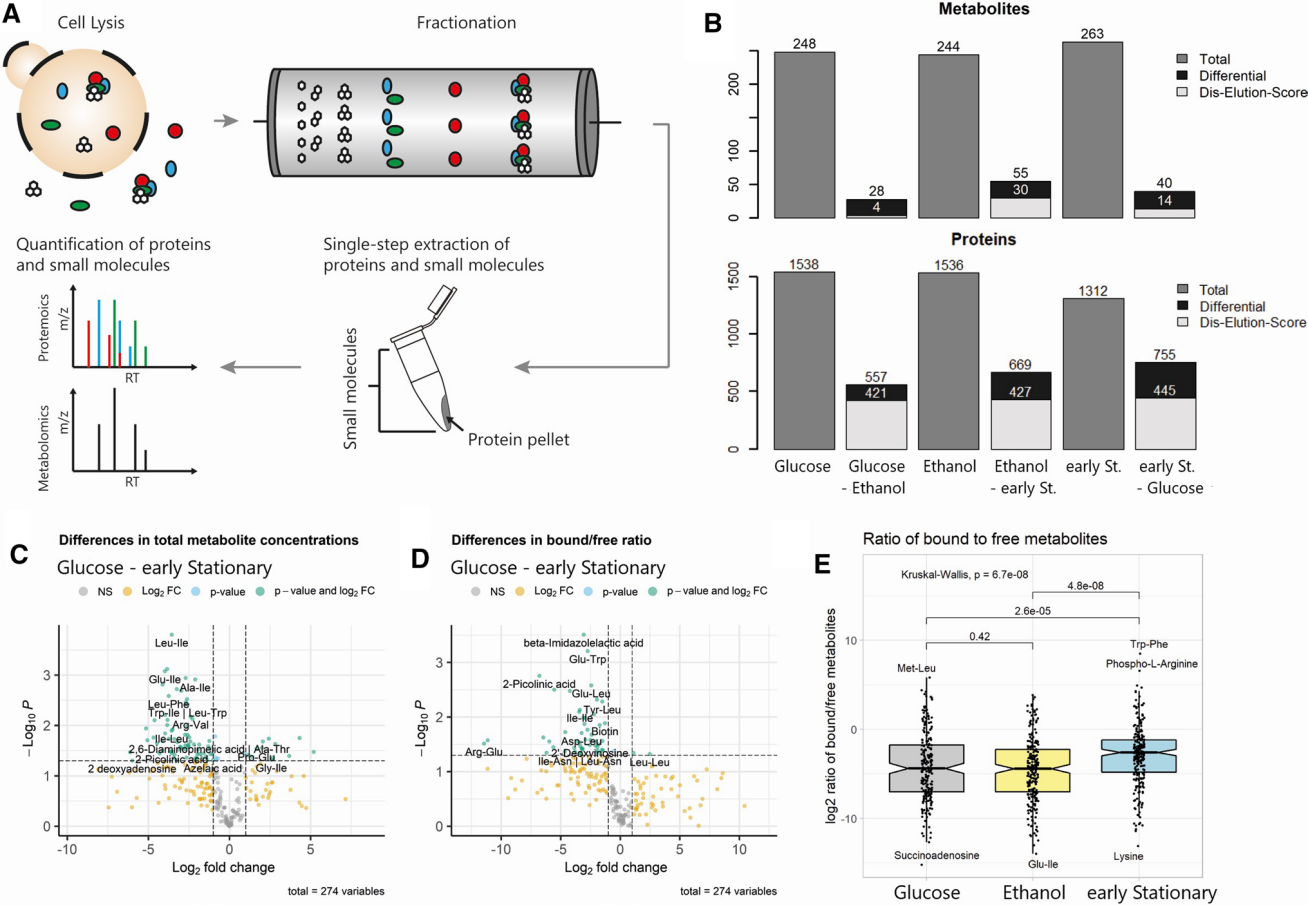
Growth phase-dependent differential fractionation of protein–metabolite complexes. **A** Schematic workflow of PROMIS: Endogenous protein—small-molecule complexes were extracted and fractionated using size exclusion chromatography. Proteins and small molecules were extracted from SEC fractions using an organic solvent-based method, which denatures proteins (resulting in formation of protein pellets) and releases small molecules from binding pockets. Proteins and small molecules were separately analysed using LC–MS. Metabolites were analysed in biological triplicates; proteins were measured in one replicate. **B** Number of differentially fractionating small molecules (upper panel) and proteins (lower panel) between the growth phases. Differentially fractionating small molecules were identified using the dis-elution score (light grey, [55] and differences in presence/absence (dark grey). For proteins, a simplified approach was used comparing the Manhattan distance of a protein to the median Manhattan distance of all proteins between two growth phases (see Materials and Methods). Early St.—early stationary. **C** Volcano plot showing the differences in total metabolite abundance between glucose-utilizing and early stationary phases. Total abundances were estimated as the sum of the metabolite fractionation profile. **D** Changes in metabolite interaction status between glucose-utilizing and early stationary phases as measured as the ratio between metabolite abundances in protein-bound and protein-free fractions. **E** Boxplot comparing the log_2_-transformed bound/free ratio of metabolites in each of the three growth phases. In the early stationary phase, metabolites show a higher fraction of bound metabolites (14.7%) compared to the glucose-utilizing phase (1.9%) and ethanol-utilizing phase (2.6%, *p* < 0.001)

The yeast cultures harvested at the different growth phases were used to prepare native cell lysates. PP and PM complexes were separated using size exclusion chromatography (SEC), yielding 60 fractions. Forty of the collected fractions are protein containing and span a size range between 5 mDa and 20 kDa, as examined using commercial reference proteins of known size. Fractions were extracted and relative abundances of proteins and metabolites were measured using mass spectrometry-based untargeted proteomics and metabolomics (Fig. 3A). We obtained a dataset containing 2812 proteins and 275 metabolites, which we annotated using an in-house library of authentic reference compounds (Tables S4 and S8, Figs. S4–S5). Elution profiles were normalized, deconvoluted and correlated using PROMISed, a novel web-based tool to facilitate analysis and visualization of the molecular interaction networks from (CF-MS) experiments [55].

First, to understand the relation between changes in metabolite abundance and PMIs, we calculated the total amount of each metabolite by summing the relative intensity across the 60 chromatographic fractions, whereas the 40 protein-containing fractions represent the protein-bound pool and the 20 protein-free fractions represent the free, unbound pool. Comparison of the total amounts revealed major differences between the growth phases, specifically, comparison of the early stationary and glucose phases revealed 72 increased and 12 decreased metabolites (FDR < 0.05 and FC > 2 or < 0.5) (Fig. 3C, Fig. S6, Table S5). The majority of accumulating metabolites were proteogenic dipeptides, in line with our previous study where we reported that proteinogenic dipeptides start to accumulate prior to the diauxic shift transition and remain high in ethanol grown yeast [36]. An interesting exception is a subgroup of proline-containing dipeptides such as Pro-Glu, which does not change or even decreases upon glucose depletion (Fig. S7). Also decreasing in abundance are nucleotides 2’-AMP, 5’-AMP, 5’-GMP, 5’-GDP, 5’-UMP and 5’-UDP. The exception being, 5’-ADP, which significantly increased in abundance in the ethanol-utilizing phase by 2.7-fold (in comparison to glucose-utilizing phase), and then drastically decreased again in the early stationary phase by 13.8-fold (in comparison to ethanol-utilizing phase).

Changes in metabolite concentrations can drive non-covalent protein–metabolite interactions; the concentration at which half of the protein is occupied is defined as binding affinity. Binding affinity also depends on a protein's status, such as presence or absence of PTMs or oligomerization state. To understand whether metabolite binding affinities globally change during the diauxic shift, we calculated the ratio between the bound and free pool of each metabolite across the three growth phases (Fig. 3E, Fig. S5, Table S5). Whereas the median ratio for glucose and ethanol phases is approximately 0.019 and 0.026, respectively, it significantly increases in the early stationary phase to 0.147 (*p* < 0.001). Among the 48 metabolites, which have a significantly higher bound to free ratio (FDR < 0.05 and FC > 2 or < 0.5, Fig. 3D) in the early stationary versus glucose phase are mainly dipeptides and nucleotides. Additionally, we find the niacin precursor kynurenine and the amino acid leucine. This increase in retention of the identified metabolites in the protein-containing fractions is highly intriguing in terms of both mechanism and function. Metabolite binding is known to enhance protein stability and in that way has protein protective properties [63], which may be especially important under conditions associated with the accumulation of damaged proteins and protein aggregates as encountered, e.g. during ageing [53].

Next, we mined PROMIS datasets for proteins and metabolites that differ in their elution profiles across the three growth phases. Since the fractionation profile of a metabolite is dependent on its protein interaction partners, a change in PMIs between the growth phases would be reflected in the metabolite´s profile. We therefore used a statistical workflow for the pairwise comparison of fractionation profiles, dubbed dis-elution score [55] as well as the presence or absence of a metabolite in a given growth phase, to identify metabolites whose fractionation profiles differ across the examined growth phases (Fig. 3B, Tables S7, S9). Our analysis identified 77 metabolites that differed in their elution profile in at least one comparison. The list comprised dipeptides, amino acids, nucleotides, co-factors and metabolic intermediates, such as kynurenine and methionine sulfoxide. This differential elution can be driven by multiple factors, such as a change in a metabolite and protein concentration, or a change in a protein oligomerization, interaction or PTMs status.

In summary, our analysis identified tens of metabolites and hundreds of proteins that differ in their elution profile across the diauxic shift transition attesting to the significant changes to the PP and PM interactomes.

### The diauxic shift is accompanied by changes in protein interaction status

To identify differentially fractionating proteins, we applied a simplified approach, in which, for each protein, we calculated the Manhattan distances between the growth phases and compared it to the median of all Manhattan distances obtained this way. A protein with a distance of at least 1.5 times the median distance was considered differential. The cut-off was based on the manual inspection of the differential elution profiles. We found that, with 755, the highest number of differential fractionating proteins were found between the glucose-utilizing and the early stationary phase, compared to the 557 between glucose and ethanol and 669 between the ethanol and early stationary phases. Since the numbers of differential proteins identified with the simplified dis-elution score (see “Materials and methods”) were similar (421, 427, 445, respectively), we attribute the large differences to the lower number of identified proteins in the early stationary phase. Taken together, this indicates that the protein oligomerization states change dramatically in the course of the diauxic shift, hinting towards global changes in the protein interaction landscape.

### Comparison of thermal stability and differential fractionation

**Fig. 4:**
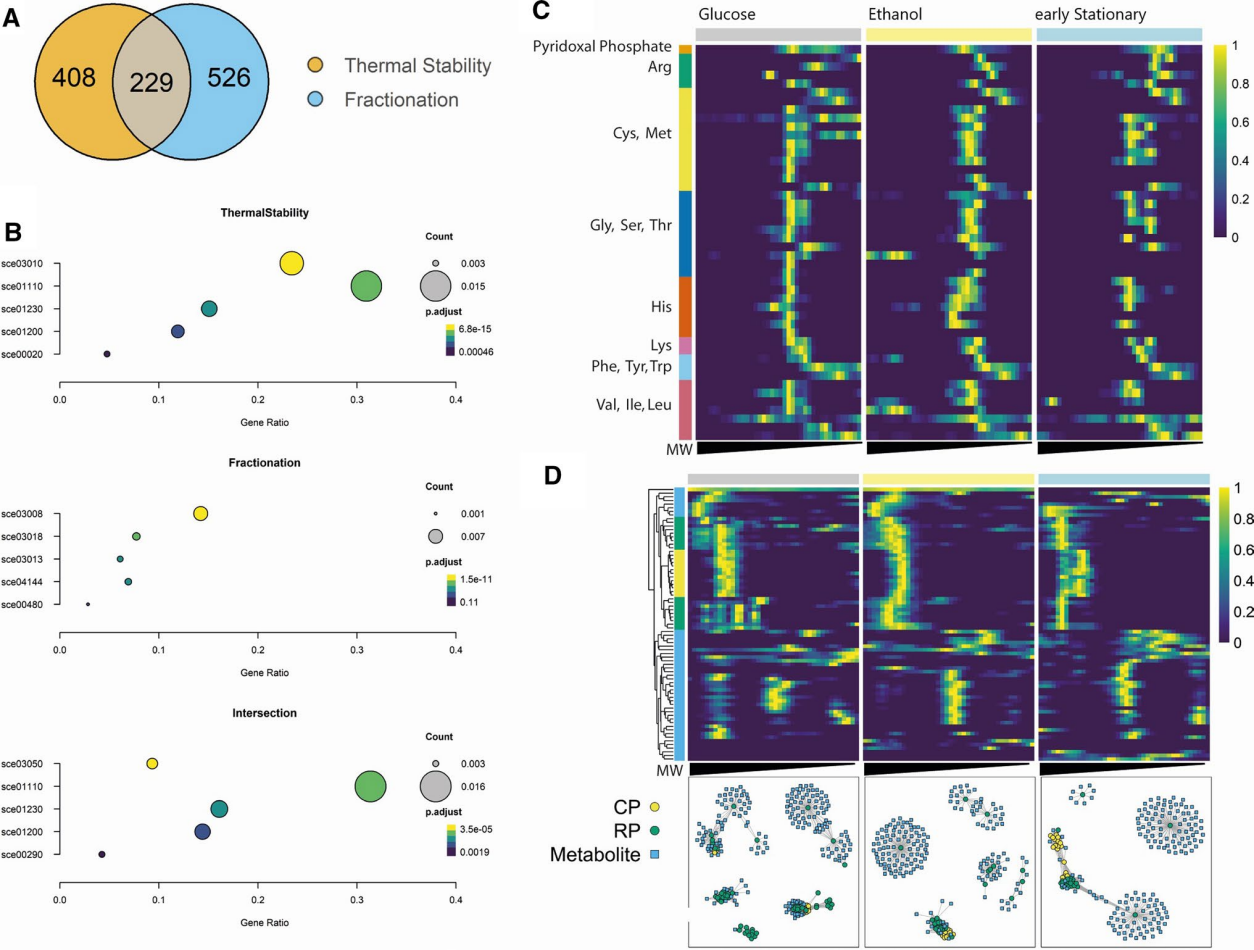
Migration pattern of core and regulatory particles of the 26S proteasome. **A** Venn diagram showing the overlap of proteins with significant changes in thermal stability and differential fractionation. **B** Top five most significant enriched KEGG pathways of the three Venn diagram sections. KEGG identifiers: sce03010: Ribosome, sce01110: Biosynthesis of secondary metabolites, sce01230: Biosynthesis of amino acids, sce01200: Carbon metabolism, sce00020: Citrate cycle (TCA cycle), sce03008: Ribosome biogenesis in eukaryotes, sce03018: RNA degradation, sce03013: Nucleoplasmic transport, sce04144: Endocytosis, sce00480: Glutathione metabolism, sce03050: Proteasome, sce00290: Valine, leucine and isoleucine biosynthesis. **C** Normalized fractionation profiles of pyridoxal phosphate (top row) and proteins involved in various amino acid metabolic pathways obtained for the glucose-utilizing, ethanol-utilizing and early stationary phases. Theoretical molecular weight was calculated using reference proteins of known mass. Proteins involved in more than one pathway are included multiple times. **D** Disassembly of the proteasome in the early stationary phase: Heatmap showing fractionation profiles of 13 proteasomal core particle (CP) proteins and 19 regulatory particle (RP) proteins, as well as 44 co-fractionating metabolites across the growth phases. Relative protein intensities were normalized to the maximum intensity of the fractionation profile. Theoretical molecular weight, ranging from 20 kDa to 5 mDa, was calculated using reference proteins of known mass. Correlation networks of subunits of the proteasomal core particle (yellow), regulatory particle (green) and co-fractionating metabolites (blue). Only correlations of 0.7 or higher are displayed

We next compared the proteins showing differential thermal stability and fractionation, focusing on the comparison between the glucose and early stationary phase. 229 proteins were characterized by the differential fractionation pattern and altered thermal stability; 408 proteins were only affected in their thermal stability and 526 proteins in their elution profile (Fig. 4A, Table S13). The 229 proteins found in the overlap were interpreted as part of dynamic protein complexes, as exemplified by the varying assembly of the proteasome (see below). The 408 proteins with differential thermal stability only were interpreted as undergoing changes in their PTM status or interactions with metabolites, or both, that do not change their oligomeric status. These were significantly enriched for proteins associated with the ribosome, secondary metabolism and biosynthesis of amino acids (Fig. 4B), including glycine, serine and threonine metabolism, such as CYS3 (see below, Table S13).

Whereas the change in the melting stability but not the fractionation pattern can be explained by the change in a ligand concentration or a protein PTMs status (discussed above), the opposite is harder to interpret. Presented here includes 526 proteins which show differential fractionation, despite being not affected in their thermal stability (Fig. 4A). This group is significantly enriched for proteins involved in ribosome biogenesis, RNA degradation, nucleocytoplasmic transport and endocytosis (Fig. 4B), and enzymes of the central carbon metabolism. There are many factors that affect thermal stability, and therefore susceptibility to thermal proteome profiling [31]. As discussed in the previous section protein length and thermal stability show a generally weak, but statistically highly significant negative correlation [31, 32, 38]. We therefore hypothesized that proteins with differential fractionation, but similar thermal stability, are, on average, smaller than other proteins identified in both experiments. However, the opposite is the case: We found that, with a median length of 468.5 aa, proteins with differential fractionation are significantly larger than proteins which are only affected in their thermal stability (median length = 332.5 aa, *p* = 1.4e12) or the median of all proteins shared between both experiments (median length = 423 aa, *p* = 0.0016, Fig. S8), and also larger than the reported median length of yeast proteins of 379 aa [11]. A much simpler explanation is that PROMIS is better suited to select even a relatively minor change of a protein oligomeric state. A single protein can exist in multiple oligomeric states, reflected by multiple maxima in the PROMIS elution profile. The distribution of a protein between the different maxima will vary and in our analysis, we accept all the maxima above the 10% of the main maxima. In contrast, because TPP/iTSA gives average stability of all the oligomeric states by default, it will mainly reflect the stability of a dominant oligomeric state and hence may miss interactions. Therefore, TPP and CF-MS are complementary approaches and can be used in combination to unravel dynamics in protein–protein interactions that would be missed using one approach alone.

### Regulation of amino acid metabolism by dynamic PMIs

Among the 408 proteins affected by thermal stability only, we found the enzyme cystathionine gamma-lyase 3 (CYS3), which catalyses the formation of cysteine from cystathionine [17, 47, 48]. We queried our dataset for metabolites interacting with CYS3 and found pyridoxal phosphate (PLP), the known co-factor of CYS3 [41]. Figure 4C displays a heatmap of fractionation profiles of PLP and proteins involved in amino acid biosynthesis that require PLP as a co-factor. PLP shows a statistically significant differential fractionation pattern between the glucose-utilizing phase and ethanol-utilizing phase (dis-elution score (DES): 9.2e–04) and between the ethanol and early stationary phases (DES: 5.29e–07), which is not the case for CYS3 and other proteins involved in amino acid metabolism (Fig. 4C). In the glucose-utilizing phase, CYS3 and PLP co-fractionate (Pearson correlation = 0.922) with a peak at around 172 kDa, the size of enzymatically active CYS3 tetramer [41]. In the early stationary phase, CYS3 shows an additional, smaller peak, corresponding to the size of the dimer at ~ 85 kDa. The PLP elution maxima shifts to even smaller sized fractions, showing no co-fractionation with CYS3 (Pearson = 0.52 for the larger peak, 0.11 for the smaller peak). This loss of interaction is accompanied by an observed decrease in thermal stability of CYS3 in the early stationary phase.

An important driver of protein–metabolite interactions is the concentration of a protein and its ligand. However, both PLP and CYS3 show no significant differences in abundance across the course of the diauxic shift. Another factor regulating PMIs are PTMs, and it has been shown that protein thermal stability can be affected by PTMs [30], especially of the phosphosites that affect protein structure [49, 56]. In human, the cystathionine gamma-lyase, CTH, is phosphorylated by PKG1-β. Yeast CYS3 and human CTH share a 51% protein sequence identity [41], and the region around CTH Ser377 and CYS3 Ser372 shows a conserved sequence. Moreover, NetPhos 3.1 [7, 8] predicts a phosphosite at Ser372 of CYS3. Therefore, we hypothesized that the interaction between CYS3 and PLP might be regulated by a dynamic phosphorylation of CYS3. However, in a recent study tracking the phosphorylation states of yeast proteins during the diauxic shift, CYS3 did not show phosphorylation at Ser372, but at Ser40. Contrary to our hypothesis, the phosphorylation state at Ser40 showed no changes in the course of the diauxic shift [23]. Therefore, the observed “loss of interaction” with PLP cannot be explained by a dynamic phosphorylation status. However, phosphorylation is not the only PTM, and the dynamic regulation of CYS3-PLP interaction could be mediated by, e.g. acetylation or methylation, presumably at the PLP binding Lys203, or at distant sites, conferring a conformational change to the enzyme.

### Reorganization of the proteasome across the diauxic shift

As mentioned above, the 229 proteins found in the overlap of iTSA and PROMIS contain multiple proteasomal proteins (Fig. 4B), specifically 13 of the 14 core particle (CP), and 18 of the 21 regulatory particle (RP) subunits. To learn about the re-arrangement of the proteasome across the diauxic shift transition we used PROMISed [55] to create interaction networks restricted to proteasomal subunits and co-fractionating metabolites (PCC > 0.7) (Fig. 4D). We used the Louvain method [9] to detect communities within the networks and obtained seven, six and five clusters, for the glucose, ethanol and early stationary phase, respectively (Fig. 4D, Tables S14–S18). We interpret these clusters as stable or transient sub-assemblies of proteasomal subunits and co-eluting metabolites. The clusters can be divided into major clusters containing multiple subunits that correspond to large complexes and minor clusters composed of few or even single subunits that correspond to small complexes or monomeric proteins.

In the glucose-utilizing phase there are two major clusters, one containing the 13 CP and 16 RP subunits and 23 metabolites and the second consisting of 17 RP subunits and 14 metabolites. The remaining five minor clusters contain up to 5 RP subunits and dozens of metabolites. In the ethanol-utilizing phase, we again identified two major clusters, one containing the 13 CP and 3 RP subunits and 4 metabolites and the second consisting of 17 RP subunits and 11 metabolites. The remaining four minor clusters contain between one and four RP subunits. Finally, in the early stationary phase, two major clusters were found, one is made up of 19 CP, a single RP subunit and five metabolites. The other cluster consists of 19 RP and 5 CP subunits and 10 metabolites. Three minor clusters contain a single RP subunit and several metabolites.

There are three observations that can be made from the network analysis. First, the number and size of the minor clusters decrease in the ethanol and early stationary phases. Second, the interaction between the two sub-complexes, CP and RP, is changing. In the glucose-utilizing phase, the majority of CP and RP subunits are clustered together. In contrast, both sub-complexes form largely separate clusters in the early stationary phase. The ethanol-utilizing phase represents an intermediate behaviour, where RP and CP form distinctive clusters, which are tightly connected, based on their co-fractionation profiles. Third, minor clusters contain more metabolites (up to 89) than major protein clusters (up to 23).

Our findings are in line with Bajorek and colleagues, who reported the disassembly of the yeast 26S proteasome (CP and RP) into the 19S (RP) and 20S (CP) sub-complexes in the stationary phase [3]. Moreover, in Arabidopsis, proteins involved in the RP and CP, respectively, possess different extremes of thermostability, in that the CP is highly stable, while the RP is a thermo-labile complex, hinting towards a higher degree of conformational flexibility of the RP [66]. In human cells under hypoxia, the CP can act as a stand-alone 20S proteasome independently of the RP, and shows distinct features compared to the human holoenzyme, as it is able to rapidly degrade unstructured proteins, generates longer peptides resulting from a distinct cleavage pattern and degrades conjugated ubiquitin instead of releasing it from the substrate [51]. Against this background we speculate that in the early stationary phase CP functions as stand-alone 20S proteasome degrading unstructured, aggregated proteins that accumulate in the ageing yeast cells. An additional mechanism to the discussed above global increase in the metabolite retention in the protein complexes.

### Dipeptides and central carbon metabolism

Proteogenic dipeptides are a recently discovered class of metabolites with mostly unknown functions. In our previous work in yeast, we showed that the dipeptide Ser-Leu interacts with and activates the glycolytic enzyme phosphoglycerate kinase (Pgk1) by increasing the enzyme's affinity to ATP [36]. A different dipeptide, Tyr-Asp, was previously shown to interact with the glycolytic enzyme glyceraldehyde 3-phosphate dehydrogenase (*Ath*GAPC) in the model plant Arabidopsis [65]. This interaction promotes tolerance to oxidative stress by redirecting carbon flux into the pentose phosphate (PPP) pathway and increasing NADPH levels [43]. Dipeptides originate from protein degradation, and, although they are always present in the cell, they accumulate in conditions associated with high rates of protein clearance, such as in response to stress [15, 36, 45, 58, 60, 71, 73]. We, for instance, found that in plants, dipeptide levels increase in response to heat and dark stresses in an autophagy-dependent manner [60], and that specific dipeptides display diurnal oscillation in response to the change in a plant carbon status, downstream of TOR signalling [12]. In comparison, yeast accumulate dipeptides in response to glucose depletion *prior* to the diauxic shift transition [36].

**Fig. 5:**
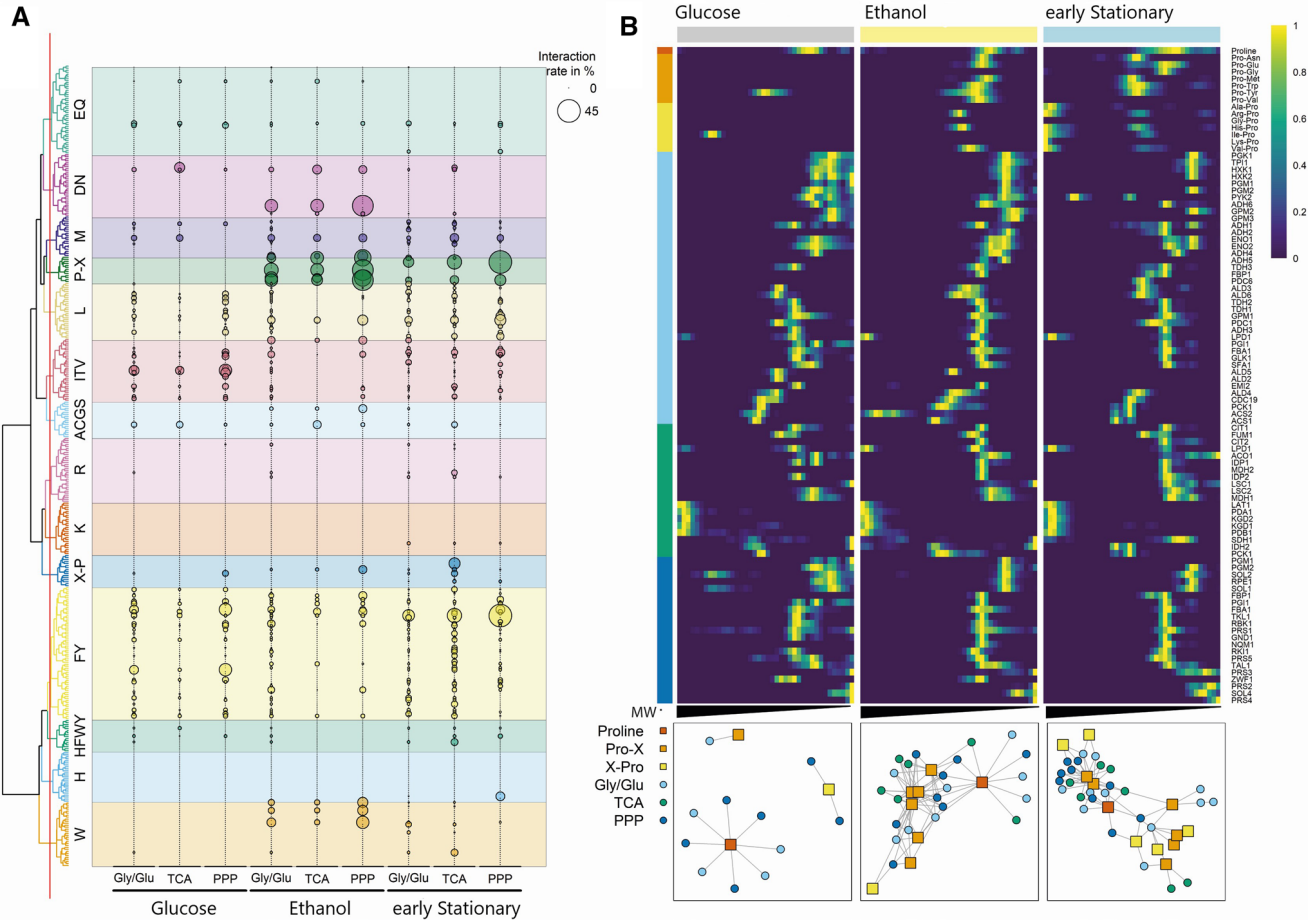
Changes in interaction rates between dipeptides and the central carbon metabolism before and after the diauxic shift. **A** Interaction rates were calculated as the percentage of observed interactions in all possible interactions between the groups and are shown as size-scaled circle. Circle colour corresponds to the dipeptide group. Proteins were grouped into Glycolysis/Gluconeogenesis (Gly/Glu), tricarboxylic acid cycle (TCA) and pentose phosphate pathway (PPP). Dipeptides were clustered based on their chemical structure similarity, resulting in 14 groups: EQ: Glu- or Gln-containing, DN: Asp- or Asn-containing, M: Met-containing, P-X: N-terminal Pro-containing, L: Leu-containing, ITV: Ile-, Thr-, or Val-containing, ACGS: Ala-, Cys-, Gly- or Ser-containing, R: Arg-containing, K: Lys-containing, X-P: C-terminal Pro-containing, FY: Phe- or Tyr-containing, HFWY: N- and C-terminal His-, Phe-, Trp- or Tyr-containing, H: His-containing, W: Trp-containing. Original data are given in Supplementary Table S19. **B** Proline-containing dipeptides co-fractionate with proteins of the central carbon metabolism in the ethanol-utilizing and early stationary phase. Top panel: Heatmap showing the normalized fractionation pattern of proline, N-terminal Pro-containing (Pro-X), C-terminal Pro-containing (X-Pro) dipeptides and proteins involved in Gly/Glu, TCA and PPP. Theoretical molecular weight, ranging from 20 kDa to 5 mDa, was calculated using reference proteins of known mass. Lower Panel: Correlation network of proline and proline-containing dipeptides and proteins involved in central carbon metabolism. Only correlations of 0.7 or higher are displayed

Given the major metabolic rewiring associated with the diauxic shift and the demonstrated role of dipeptides in regulating central carbon metabolism, we queried the current datasets for changing dipeptide–enzyme interactions. Among the 274 annotated protein-bound metabolites 145 (53%) are proteogenic dipeptides. To get a better understanding of the protein–dipeptide interaction network, we first grouped the 400 proteogenic dipeptides using the ChemmineR package [18], resulting in 14 dipeptide groups (Fig. 5A). Four groups are composed of aromatic dipeptides, and 10 of non-aromatic dipeptides. We also grouped the enzymes of the central carbon metabolism into three categories based on the KEGG annotations: glycolysis/gluconeogenesis (Gly/Glu), tricarboxylic acid cycle (TCA) and the pentose phosphate pathway (PPP). We then calculated the interaction rate between dipeptides and enzymes of the different pathways as the percentage of observed interactions in the total of possible interactions (Fig. 5A, Table S19 + S20).

The group of dipeptides that stood out was the N’-terminal proline-containing dipeptides (P-X) (Fig. 5A, B). These were almost entirely absent in the protein-containing fractions in the glucose-utilizing phase with only one putative interaction. This is in stark contrast to ethanol and early stationary phases. The number of co-fractionating proteins and resulting interactions increased to 17 and 69, in the ethanol-utilizing phase, and to 26 proteins and 48 interactions in the early stationary phase. This increase in interactions cannot be attributed to changes in P-X dipeptide concentration, since the levels of P-X dipeptides do not change or even decrease upon glucose depletion. Enzymes co-fractionating with P-X include glyceraldehyde-3-phosphate dehydrogenase (TDH1, TDH2 and TDH3), fructose 1,6-bisphosphate aldolase (FBA1), phosphoglycerate mutase (GPM1), 3-phosphoglycerate kinase (PGK1) and citrate synthases (CIT1 and CIT2) (Table S21 + S22). Interestingly, a similar list of putative interaction partners of proline-containing dipeptides has been reported in *Arabidopsis thaliana* [12], pointing to the putative conserved role of proline-containing dipeptides in the regulation of central carbon metabolism.

## Conclusions

Here, we combined two approaches, iTSA and PROMIS, to investigate proteome- and metabolome-wide changes in the PPI and PMI landscape during the transition from fermentative to respiratory growth in *Saccharomyces cerevisiae*. *On par* with previous studies, e.g. Murphy et al. [44] we reported hundreds of proteins and tens of metabolites accumulating differentially across the diauxic shift transition. Most importantly, the reported changes translate into a significant rewiring of the protein–metabolite interactome,the most pronounced difference measured between glucose and early stationary phases. Intriguingly, global analysis of the protein-bound to free metabolite ratios revealed that small molecules are preferentially retained in the protein complexes during early stationary phase. We speculate it may be related to the metabolite-driven proteoprotection [63], particularly that entry into the stationary growth phase is associated with accumulation of aggregated proteins [46].

There is only a 20% overlap of the differential proteins characterized by the change in both the thermal stability and elution profile. Thus, combining both methods is better to obtain a comprehensive view of the differential interactome. Whereas iTSA reveals a major change in a protein interaction or PTM status, PROMIS can capture the different protein oligomerization states and also provide a glimpse into the nature of the interacting partners, including small molecules. We demonstrate that CF-MS, and specifically PROMIS, is suitable to study the dynamics of protein–metabolite complexes across multiple cell states. The biggest challenge for any CF-MS method is to distinguish between the true and coincidental co-elution, and hence to identify true interactors. We expect that analogously to what was done for the PPIs, we will be able to significantly improve the identification of true protein–metabolite complexes by combining multiple datasets and chromatographic methods, increasing the number of collected fractions and by introducing machine learning approaches building on the known interactions, reviewed by Wagner et al. [67].

The present dataset attests to the dynamic rewiring of metabolite–protein–protein complexes accompanying the switch from fermentative to respiratory growth in yeast. It provides a valuable resource for unravelling the role of PPI and PMI in adjusting yeast metabolism to the changes in glucose availability. We present three examples of the differential PP and PM interactions that we think constitute novel regulatory mechanisms governing the diauxic shift. (i) Pyridoxal phosphate (PLP) is a known co-factor of enzymes involved in transamination [61, 62]. Amino acid homeostasis relies on three principal processes: amino acid uptake, de novo synthesis and recycling. During starvation (stationary phase) yeast mostly recycles amino acids through proteolysis [1]. Here, we report a loss of co-elution and hence a change in binding, between amino acid biosynthetic enzymes, exemplified by CYS3, and PLP in the early stationary phase. We speculate that the “loss of PLP binding” may be due to a change in the PTMs status, other than phosphorylation, which would lead to the inhibition of the enzymatic activity, contributing to the downregulation of de novo amino acid synthesis in the early stationary phase. (ii) Next, we report a gradual disassembly of the 26S proteasome into the 20S (CP) and 19S (RP) sub-complexes associated with the switch between fermentative and respiratory growth. Our observations are in line with previous literature findings that showed a disassembly of the proteasome into stable complexes, accompanied by a reduction in proteolytic activity [3]. Nevertheless, the CP is able to degrade its substrates independently of the RP in various eukaryotic models [51]. Against this background we speculate that in the early stationary phase CP functions as stand-alone 20S proteasome degrading unstructured, aggregated proteins that accumulate in the ageing yeast cells. (iii) Lastly, and on par with our previous report from plants [12], we demonstrate a co-elution, and hence a putative interaction, between the proline-containing, Pro-X, dipeptides, and various enzymes of the central carbon metabolism specifically in the early stationary phase. Intriguingly the appearance of Pro-X dipeptides in the protein complexes is independent of the cellular concentrations pointing to the binding being dependent on, e.g. a change in a protein(s) PTMs status. And, however, the identity of the Pro-X protein targets needs to be independently validated; it solidifies the shown before regulatory roles of dipeptides [36, 43, 45, 72], beyond simply intermediates of the protein degradation.

In summary, (i) the present dataset attests to the dynamic rewiring of metabolite–protein–protein complexes accompanying the switch from fermentative to respiratory growth in yeast. It provides a valuable resource for unravelling the role of PPI and PMI in adjusting yeast metabolism to the changes in glucose availability. Notably, whereas the switch between fermentative to respiratory growth is also of interest to cancer metabolism research, the entry into the stationary phase shares molecular signatures with chronological ageing in other organisms, including humans. (ii) Moreover, we demonstrate that CF-MS, and specifically PROMIS, is suitable to study the dynamics of protein–metabolite complexes across multiple cell states. Finally, as discussed before [36, 43] the reported small molecules represent just a small subset of all the metabolic features measured in the protein-containing fractions that we could annotate. In the future and by concentrating on the chemical identification of the “unknown” metabolic features showing differential elution, we aim to identify novel small-molecule regulators of the diauxic shift transition.

## Methods

### Yeast growth and cell lysis

Experiments were performed using the YSBN2 strain of *Saccharomyces cerevisiae* ordered from the EUROSCARF (strain number: Y40383) cultivated in YPD medium at 30 °C with moderate shaking (120–140 RPM) using Innova Shakers. A single colony grown on a YPD plate was used to inoculate a pre-culture. Pre-culture was cultivated for 24 h. The following day, a pre-culture was used to inoculate a culture. The starting OD was 0.03. Cells were collected by centrifugation (4000 *g*, 4 °C, 20 min) after 6, 24 and 72 h of cultivation, corresponding to growth in the glucose-utilizing, early ethanol-utilizing and late ethanol-utilizing phase, respectively. Cell pellets were washed with AmBIC buffer (50 mM ammonium bicarbonate, 150 mM NaCl, 1.5 mM MgCl_2_), transferred to 50 mL tubes and centrifuged again (4000 *g*, 4 °C, 20 min). The pellets were then snap frozen in liquid nitrogen and stored at − 80 °C until further use. Collected cells were mixed with an ice-cold lysis buffer (50 mM AmBIC, 150 mM NaCl, 1.5 mM MgCl_2_, 5 mM DTT, 1 mM PMSF, 1 × cOmplete EDTA-free Protease Inhibitor Cocktail (MERCK, 11873580001), 0.1 mM Na3VO4 and 1 mM NaF) and frozen (− 20 °C) silica-zirconia beads (Biospec, 11079105z). Yeasts were homogenized by bead beating 10 × 30 s at 20 Hz using a Retsch Mixer Mill MM 400 and cooled in an ice water bath for 1 min in-between bead beating. Cell debris and silica-zirconia beads were sedimented by a 10 min centrifugation at 4000 *g* and 4 °C. After centrifugation, the supernatant was used for either the Isothermal Shift Assay or PROMIS.

### Isothermal shift assay

The protocol was adapted from Ball et al. [4]. The supernatant (see above) was transferred to Eppendorf tube and centrifuged for 10 min at 21,000 *g* and 4 °C. The protein concentration of the supernatant was determined using the Bradford assay. Equivalent of 200 µg of proteins (5 replicates for each of the 3 growth phases) was transferred to PCR tubes and heated to 48 °C, 52 °C or 56 °C for 3 min and then incubated for 3 min at room temperature. During this time samples were moved to 1.5 mL tubes and centrifuged for 20 min at 21,000 *g* and 4 °C. The supernatant was then transferred to fresh Eppendorf tubes and proteins were precipitated overnight at − 20 °C using 80% Acetone (MS-grade). Next day, proteins were pelleted by centrifugation for 20 min at 21,000 *g* and 4 °C. Supernatant was removed and proteins were air-dried. Finally, proteins were digested and desalted as described in section LC–MS/MS of proteins. Dried peptides were suspended in 60 µL MS loading buffer (2% ACN, 0.2% TFA), separated and quantified using LC–MS as described in section LC–MS/MS of proteins.

### Separation of endogenous complexes using size exclusion chromatography

The protocol was adapted from Luzarowski et al. [36]. The supernatant (see above) was transferred to ultracentrifuge tubes and centrifuged for one hour at 35,000 RPM (max. 148,862 *g*, avg. 116,140 *g*) at 4 °C to obtain the soluble fraction containing endogenous complexes. The soluble fraction was loaded onto previously pre-rinsed (15 mL wash buffer: 50 mM AmBIC, 150 mM NaCl, 1.5 mM MgCl_2_, 20 min, 4000 *g*, 4 °C) Amicon Ultra-15 centrifugal filter units (10 kDa MWCO) and centrifuged for 20 min at 4000 *g*, 4 °C.

Soluble fraction, corresponding to 40 mg of protein, was loaded on Sepax SRT SEC-300 21.2 × 300 mm column (Sepax Technologies, Inc., Delaware Technology Park, separation range 1.2 mDa to 10 kDa) connected to an ÄKTA explorer 10 (GE Healthcare Life Science, Little Chalfont, UK) and separated at 7 mL/min flow rate, 4 °C. 50 mM AmBIC pH 7.5, 150 mM NaCl and 1.5 mM MgCl2 was used to equilibrate the column and separate molecular complexes. Forty 1-mL fractions were collected from the 39 mL to 78 mL elution volume. The fractions were frozen by snap freezing in liquid nitrogen and subsequently lyophilized and stored at − 80 °C for metabolite and protein extractions.

### Extraction of proteins and polar metabolites

The extraction protocol was adapted and modified from [57]. Proteins and metabolites were extracted from the lyophilized fractions using a methyl tert-butyl ether (MTBE)/methanol/water solvent system. Equal volumes of the polar fraction and protein pellet were dried in a centrifugal evaporator and stored at − 80 °C until they were processed further.

### LC–MS/MS of proteins

Fractionated proteins were quantified using the Bradford assay. Protein pellets from 40 fractions corresponding to 39–78 mL elution volume were suspended in 30 µL urea buffer (6 M urea, 2 M thiourea in 40 mM ammonium bicarbonate). 20 µg of proteins from each fraction was reduced, alkylated and enzymatically digested using LysC/Trypsin Mix (Promega Corp., Fitchburg, WI) according to the manufacturer’s instructions. Self-made C18 Empore^®^ extraction disks (3 M, Maplewood, MN) STAGE tips were used for protein desalting [50]. Proteins were concentrated using the centrifugal evaporator to approximately 4 µL and stored at − 80 °C until measured. Dried peptides were suspended in 60 µL MS loading buffer (2% ACN, 0.2% TFA), and 3 µL (equivalent to 0.8–1.0 µg of peptides) were separated using C18 reversed-phase column connected to an ACQUITY UPLC M-Class system in a 120 min gradient. The gradient started from 3.2% and increased to 7.2% ACN in 20 min, to 24.8% ACN over 70 min and to 35.2% ACN over 30 min, followed by a 5 min washout with 76% ACN. The Thermo Q Exactive HF operated with a data-dependent method as follows: MS full scans were performed in FTMS with resolution set to 120,000, from 300.0 to 1600.0 *m*/*z*, a maximum fill time of 50 ms and an AGC target value of 3e6 ions. A maximum of 12 data-dependent MS2 scans was performed in the ion trap set to an AGC target of 1e5 ions with a maximal injection time of 100 ms. Precursor ion fragmentation was achieved with collision-induced fragmentation with a normalized collision energy of 27 and isolation width of 1.2 *m*/*z*. Charge states of 1 and ≥ 7 were excluded.

### LC–MS metabolomics

After extraction, the dried aqueous phase was measured using ultra-performance liquid chromatography coupled to a Q-Exactive mass spectrometer (Thermo Fisher Scientific) in positive and negative ionization modes, as described earlier [25].

### Data pre-processing: LC–MS metabolite data

Expressionist Refiner MS 12.0 (Genedata AG, Basel, Switzerland) was used for processing the LC–MS data with the following settings. Repetition was used to reduce the volume of data and to speed up processing. All types of data except Primary MS Centroid Data were removed using Data Sweep. Chemical Noise Subtraction activity was used to remove artefacts caused by chemical contamination. Snapshot of chromatogram was saved for further processing. Further processing of chromatogram snapshot were performed as follows: chromatogram alignment (RT search interval 0.5 min), peak detection (minimum peak size 0.03 min, gap/peak ratio 50%, smoothing window 5 points, centre computation by intensity-weighted method with intensity threshold at 70%, boundary determination using inflection points), isotope clustering (RT tolerance at 0.02 min, *m*/*z* tolerance 5 ppm, allowed charges 1–4), filtering for a single peak not assigned to an isotope cluster, charge and adduct grouping (RT tolerance 0.02 min, m/z tolerance 5 ppm). A more detailed description of the software usage and possible settings was published before [57]. In-house library of authentic reference compounds was used to identify molecular features allowing 0.005 Da mass deviation and dynamic retention time deviation (maximum 0.2 min). Processing of fractionated samples resulted in annotation of 282 small molecules across three growth phases.

### Data analysis

Protein data analysis was restricted to the 1627 proteins commonly identified across all experiments.

### Analysis of protein abundance

Protein intensities were measured in three growth phases in five replicates. Protein intensities were normalized to the median intensity across all samples as Int_norm_ = Int_protein_ * (Median_sample_/Median_global_). To identify differences in protein abundance between growth phases in a pairwise manner, we calculated the fold changes and performed a two-sided Student's t-test.

### Analysis of thermal stability

After treatment with 3 temperatures (48 °C, 52 °C and 56 °C) and subsequent removal of denatured proteins by centrifugation, the abundance of soluble proteins was measured in five replicates. Protein intensities were normalized to the median intensity within each temperature group and scaled to the protein abundance at room temperature. Proteins with significant (FDR < 0.01) fold changes between growth phases in the same temperature group were considered as having a differential thermal stability. At 56 °C, 21 proteins showed a differential thermal stability between glucose-utilizing phase and ethanol-utilizing phase, 531 between glucose-utilizing phase and early stationary phase and 8 between ethanol and early stationary phases, respectively.

### Analysis of differential protein elution profiles

For each protein, the Manhattan distance (MD) was calculated between processed elution profiles of all three growth phases in a pairwise manner. From this, the median MD was determined, and proteins with a MD greater than 1.5 of the median were considered as differentially eluting.

### Protein data integration

To further narrow down the list of proteins of interest, we integrated the different datasets using the VennDiagram package [16]. We then performed KEGG enrichment analysis for proteins present in the different sections of the Venn diagram using the clusterProfiler package [74].

### Volcano plots

Volcano plots are generated using the EnhancendVolcano R package [6]. The *x*-axis shows the log_2_ fold change (FC), the *y*-axis the − log_10_ *p* value. The horizontal, dotted line indicates the *p* value threshold (0.01), the vertical, dotted lines the thresholds of fold changes (− 2 and 2). Points correspond to proteins and are coloured as follows: Grey: FC between − 2 and 2, *p* > 0.01, Green: FC outside of thresholds, *p* > 0.01, Blue: FC between thresholds, *p* < 0.01, and Red: FC outside of thresholds, *p* < 0.01. Dipeptide clustering was based on chemical structure similarity.

### Dipeptide clustering

Smiles codes of dipeptides were obtained from https://pubchem.ncbi.nlm.nih.gov/ on 12.10.2021. Pairwise distances between dipeptides were calculated on atom pair libraries using cmp.cluster() of the ChemmineR package [18]. Clustering was performed using Ward´s minimum variance method [69]. The resulting dendrogram was cut at height 1.6, resulting in 14 dipeptide groups named after the predominant amino acids.

### Calculation of interaction rates

Fractionation profiles of dipeptides and proteins involved in central carbon metabolism were analysed with PROMISed [55], using default settings, and correlation tables were obtained. PROMISed splits fractionation profiles into distinct, single peaks and calculates Pearson correlation between all obtained metabolite and protein peaks. We filtered the obtained correlation table for co-fractionating pairs using a threshold of 0.7. We then grouped all proteins based on their involvement in Glycolysis/Gluconeogenesis, tricarboxylic acid cycle (TCA cycle) and the pentose phosphate pathway (PPP). The interaction rate between dipeptides and these groups was calculated as the percentage of observed interactions in all possible interactions as

$${\text{InteractionRate }} = \, {{n_{{{\text{Interactions}}}} } \mathord{\left/ {\vphantom {{n_{{{\text{Interactions}}}} } {\left( {n_{{{\text{DipeptidePeaks}}}} *n_{{{\text{ProteinPeaks}}}} } \right)}}} \right. \kern-\nulldelimiterspace} {\left( {n_{{{\text{DipeptidePeaks}}}} *n_{{{\text{ProteinPeaks}}}} } \right)}}*100,$$

where *n*_Interactions_ is the number of observed co-fractionations (PCC > 0.7), *n*_DipeptidePeaks_ the number of peaks originating from the dipeptide and *n*_ProteinPeaks_ the number of peaks of proteins in that group.

## Supplementary Information

Below is the link to the electronic supplementary material.

Supplementary file1 Supplementary Figure 1: Volcano plots showing the differences in protein thermal stability at 48 °C (left), 52 °C (middle) and 56°C (right) between the three growth phases. Upper panel, between glucose and ethanol phase; middle panel, between glucose and early stationary phase; lower panel between ethanol and early stationary phase. The horizontal, dashed line indicates FDR = 0.01, vertical, dashed lines indicate a fold-change greater than 2. Proteins with significant (FDR < 0.01) fold changes greater than 2 are highlighted in green, and proteins involved in central carbon metabolism are labelled. (JPG 3801 KB)

Supplementary file2 Supplementary Figure 2: Protein thermal stability and protein length show a weak, but statistically significant negative correlation. Scatter plots showing the relative protein abundance after treatment at 56 °C versus protein length in amino acids. For the early stationary phase, the protein MAK21 was excluded from the analysis. (JPG 496 KB)

Supplementary file3 Supplementary Figure 3: Subunits of the macromolecular complexes identified as differential between early stationary and glucose phases in the iTSA experiment. PPIs (experimental evidence, confidence score >0.9) were downloaded from STRING database. Figure was generated using Cytoscape. 1 Ribosome, 2 Proteasome, 3 RNA polymerase complex, 4 Aminoacyl-tRNA synthetase complex, 5 Cytochrome c reductase complex, 6 Coatomer, 7 CTT chaperone complex, 8 V-ATPase (JPEG 2065 KB)

Supplementary file4 Supplementary Figure 4: Clustered heatmap of normalized protein fractionation patterns. Protein fractionation patterns of the three growth phases (grey, glucose; yellow, ethanol; blue, early stationary) are concatenated and clustered using euclidean distance and the “complete” hierarchical clustering method as implemented in the pheatmap R-pacakge. (JPG 3816 KB)

Supplementary file5 Supplementary Figure 5: Clustered heatmap of normalized metabolite fractionation patterns in the protein containing fractions. Metabolite fractionation patterns of the three growth phases (grey, glucose; yellow, ethanol; blue, early stationary) are concatenated and clustered using euclidean distance and the “complete” hierarchical clustering method as implemented in the pheatmap R-pacakge. Note that metabolites which are depicted as absent in the heatmap are present in the non-protein containing fractions, or the fractionation profiles between replicates may not be reproducible. (JPG 2584 KB)

Supplementary file6 Supplementary Figure 6: A) Volcano plot showing the differences in total metabolite abundance between glucose and ethanol phase (top), glucose and early stationary phase (middle), and ethanol and early stationary phase (bottom), respectively. Total abundances were estimated as the sum of the metabolite fractionation profile. B) Changes in metabolite interaction status between glucose and ethanol phase (top), glucose and early stationary phase (middle), and ethanol and early stationary phase (bottom), respectively, as measured as the ratio between metabolite abundances in protein bound and protein free fractions. (JPG 842 KB)

Supplementary file7 Supplementary Figure 7: Heat-map of dipeptide accumulatio across the yeast growth stages. Data are expressed as log2 fold change in comparison to the glucose-utilizing phase. (JPG 839 KB)

Supplementary file8 Supplementary Figure 8: Violin plot showing protein length in amino acids for proteins with differential fractionation only (Fractionation), differences in thermal stability only (ThermalStability), and proteins affected by either or both experiments (Shared Proteins) between the glucose and late ethanol -utilizing phase (see also Figure 4 A and Table S13). Proteins affected by fractionation only are significantly larger than proteins affected by thermal stability only. (JPG 109 KB)

Supplementary file9 (XLSX 53521 KB)

## Data Availability

Raw and analysed data can be found in the Supplementary Material. Proteomics chromatograms were deposited to PRIDE. PXD026639 [Deutsch EW, Bandeira N, Sharma V, Perez-Riverol Y, Carver JJ, Kundu DJ, García-Seisdedos D, Jarnuczak AF, Hewapathirana S, Pullman BS, Wertz J, Sun Z, Kawano S, Okuda S, Watanabe Y, Hermjakob H, MacLean B, MacCoss MJ, Zhu Y, Ishihama Y, Vizcaíno JA(2020). The ProteomeXchange consortium in 2020: enabling ‘big data’ approaches in proteomics, Nucleic Acids Res 48(D1):D1145-D1152 (PubMed PMID: 31686107).

## References

[1] Alvers AL, Fishwick LK, Wood MS, Doreen Hu, Chung SH, Dunn Jr WA, Aris JP (2009). Autophagy and amino acid homeostasis are required for chronological longevity in *Saccharomyces cerevisiae*. Aging Cell.

[2] Aryal UK, Xiong Yi, McBride Z, Kihara D, Xie J, Hall MC, Szymanski DB (2014). A proteomic strategy for global analysis of plant protein complexes. Plant Cell.

[3] Bajorek M, Finley D, Glickman MH (2003). Proteasome disassembly and downregulation is correalted with viabolity during stationary phase. Curr Biol.

[4] Ball KA, Webb KJ, Coleman SJ, Cozzolino KA, Jacobsen J, Jones KR, Stowell MHB, Old WM (2020). An Isothermal shift assay for proteome scale drug-target identification. Commun Biol.

[5] Becher I, Andrés-Pons A, Romanov N, Stein F, Schramm M, Baudin F, Helm D, Kurzawa N, Mateus A, Mackmull MT, Typas A, Müller CW, Bork P, Beck M, Savitski MM (2018). Pervasive protein thermal stability variation during the cell cycle. Cell.

[6] Blighe K, Rana S, Lewi M (2022) EnhancedVolcano: publication-ready volcano plots with enhanced colouring and labeling. R Package Version 1.8.0

[7] Blom N, Gammeltoft S, Brunak S (1999). Sequence and structure-based prediction of eukaryotic protein phosphorylation sites. J Mol Biol.

[8] Blom N, Sicheritz-Pontén T, Gupta R, Gammeltoft S, Brunak S (2004). Prediction of post-translational glycosylation and phosphorylation of proteins from the amino acid sequence. Proteomics.

[9] Blondel VD, Guillaume JL, Lambiotte R, Lefebvre E (2008). Fast unfolding of communities in large networks. J Stat Mech: Theory Exp.

[10] Broach JR (2012). Nutritional control of growth and development in yeast. Genetics.

[11] Brocchieri L, Karlin S (2005). Protein length in eukaryotic and prokaryotic proteomes. Nucleic Acids Res.

[12] Calderan-Rodrigues MJ, Luzarowski M, Monte-Bello CC, Minen RI, Zühlke BM, Nikoloski Z, Skirycz A, Caldana C (2021). Proteogenic dipeptides are characterized by diel fluctuations and target of rapamycin complex-signaling dependency in the model plant *Arabidopsis thaliana*. Front Plant Sci.

[13] Canelas AB, Harrison N, Fazio A, Zhang J, Pitkänen JP, Van Den Brink J, Bakker BM, Bogner L, Bouwman J, Castrillo JI, Cankorur A, Chumnanpuen P, Daran-Lapujade P, Dikicioglu D, Van Eunen K, Ewald JC, Heijnen JJ, Kirdar B, Mattila I, Mensonides FIC, Niebel A, Penttilä M, Pronk JT, Reuss M, Salusjärvi L, Sauer U, Sherman D, Siemann-Herzberg M, Westerhoff H, De Winde J, Petranovic D, Oliver SG, Workman CT, Zamboni N, Nielsen J (2010). Integrated multilaboratory systems biology reveals differences in protein metabolism between two reference yeast strains. Nat Commun.

[14] Chan JNY, Vuckovic D, Sleno L, Olsen JB, Pogoutse O, Havugimana P, Hewel JA, Bajaj N, Wang Y, Musteata MF, Nislow C, Emili A (2012). Target identification by chromatographic co-elution: monitoring of drug-protein interactions without immobilization or chemical derivatization. Mol Cell Proteomics.

[15] Chaudhri VK, Salzler GG, Dick SA, Buckman MS, Sordella R, Karoly ED, Mohney R, Stiles BM, Elemento O, Altorki NK, McGraw TE (2014). Metabolic alterations in lung cancer-associated fibroblasts correlated with increased glycolytic metabolism of the tumor. Mol Cancer Res.

[16] Chen H (2018) VennDiagram: Generate High-Resolution Venn and Euler Plots. R Package Version 1.6.20

[17] Cherest H, Thomas D, Surdin-Kerjan Y (1993). Cysteine biosynthesis in saccharomyces cerevisiae occurs through the transsulfuration pathway which has been built up by enzyme recruitment. J Bacteriol.

[18] Coa EY, Horan K, Backman T, Girke T (2022) ChemmineR. v. 3.14

[19] Dai L, Zhao T, Bisteau X, Sun W, Prabhu N, Lim YT, Sobota RM, Kaldis P, Nordlund P (2018). Modulation of protein-interaction states through the cell cycle. Cell.

[20] DeRisi JL, Iyer VR, Brown PO (1999). Exploring the metabolic and genetic control of gene expression on a genomic scale. Chemtracts.

[21] Duan G, Walther D (2015). The roles of post-translational modifications in the context of protein interaction networks. PLoS Comput Biol.

[22] Galdieri L, Mehrotra S, Sean Yu, Vancura A (2010). Transcriptional regulation in yeast during diauxic shift and stationary phase. OMICS.

[23] Gassaway BM, Paulo JA, Gygi SP (2021). Categorization of phosphorylation site behavior during the diauxic shift in *Saccharomyces cerevisiae*. J Proteome Res.

[24] Geladaki A, Britovšek NK, Breckels LM, Smith TS, Vennard OL, Mulvey CM, Crook OM, Gatto L, Lilley KS (2019). Combining LOPIT with differential ultracentrifugation for high-resolution spatial proteomics. Nat Commun.

[25] Giavalisco P, Li Y, Matthes A, Eckhardt A, Hubberten HM, Hesse H, Segu S, Hummel J, Köhl K, Willmitzer L (2011). Elemental formula annotation of polar and lipophilic metabolites using 13C, 15N and 34S isotope labelling, in combination with high-resolution mass spectrometry. Plant J.

[26] Gorka M, Swart C, Siemiatkowska B, Martínez-Jaime S, Skirycz A, Streb S, Graf A (2019). Protein complex identification and quantitative complexome by CN-PAGE. Sci Rep.

[27] Hammad N, Rosas-Lemus M, Uribe-Carvajal S, Rigoulet M, Devin A (2016). The crabtree and warburg effects: do metabolite-induced regulations participate in their induction?. Biochimica et Biophysica Acta - Bioenergetics.

[28] Heusel M, Frank M, Köhler M, Amon S, Frommelt F, Rosenberger G, Bludau I, Aulakh S, Linder MI, Liu Y, Collins BC, Gstaiger M, Kutay U, Aebersold R (2020). A global screen for assembly state changes of the mitotic proteome by SEC-SWATH-MS. Cell Syst.

[29] Hu LZ, Ming FG, Tan JH, Wolf E, Kuzmanov U, Wan C, Phanse S, Changjiang Xu, Schertzberg M, Fraser AG, Bader GD, Emili A (2019). EPIC: software toolkit for elution profile-based inference of protein complexes. Nat Methods.

[30] Huang JX, Lee G, Cavanaugh KE, Chang JW, Gardel ML, Moellering RE (2019). High throughput discovery of functional protein modifications by hotspot thermal profiling. Nat Methods.

[31] Jarzab A, Kurzawa N, Hopf T, Moerch M, Zecha J, Leijten N, Bian Y, Musiol E, Maschberger M, Stoehr G, Becher I, Daly C, Samaras P, Mergner J, Spanier B, Angelov A, Werner T, Bantscheff M, Wilhelm M, Klingenspor M, Lemeer S, Liebl W, Hahne H, Savitski MM, Kuster B (2020). Meltome atlas—thermal proteome stability across the tree of life. Nat Methods.

[32] Leuenberger P, Ganscha S, Kahraman A, Cappelletti V, Boersema PJ, Von Mering C, Claassen M, Picotti P (2017). Cell-wide analysis of protein thermal unfolding reveals determinants of thermostability. Science.

[33] Li X, Snyder M (2011). Metabolites as global regulators: a new view of protein regulation. BioEssays.

[34] Li Y, Kuhn M, Zukowska-Kasprzyk J, Hennrich ML, Kastritis PL, O’Reilly FJ, Phapale P, Beck M, Gavin AC, Bork P (2021). Coupling proteomics and metabolomics for the unsupervised identification of protein-metabolite interactions in *Chaetomium thermophilum*. PLoS ONE.

[35] Lindsley JE, Rutter J (2006). Whence cometh the allosterome?. Proc Natl Acad Sci USA.

[36] Luzarowski M, Vicente R, Kiselev A, Wagner M, Schlossarek D, Erban A, Perez L, de Souza D, Childs IW, Luzarowska U, Górka M, Sokołowska EM, Kosmacz M, Moreno JC, Brzezińska A, Vegesna B, Kopka J, Fernie AR, Willmitzer L, Ewald JC, Skirycz A (2021). Global mapping of protein-metabolite interactions *in Saccharomyces cerevisiae* reveals that ser-leu dipeptide regulates phosphoglycerate kinase activity. Commun Biol.

[37] Mallam AL, Sae-Lee W, Schaub JM, Fan Tu, Anna Battenhouse Yu, Jang J, Kim J, Wallingford JB, Finkelstein IJ, Marcotte EM, Drew K (2019). Systematic discovery of endogenous human ribonucleoprotein complexes. Cell Rep.

[38] Mateus A, Bobonis J, Kurzawa N, Stein F, Helm D, Hevler J, Typas A, Savitski MM (2018). Thermal proteome profiling in bacteria: probing protein state in vivo. Mol Syst Biol.

[39] Mateus A, Hevler J, Bobonis J, Kurzawa N, Shah M, Mitosch K, Goemans CV, Helm D, Stein F, Typas A, Savitski MM (2020). The functional proteome landscape of *Escherichia coli*. Nature.

[40] McWhite CD, Papoulas O, Drew K, Dang Vy, Leggere JC, Sae-Lee W, Marcotte EM (2021). Co-Fractionation/mass spectrometry to identify protein complexes. STAR Protocols.

[41] Messerchmidt A, Worbs M, Steegborn C, Wahl MC, Huber R, Laber B, Clausen T (2003). Determinants of enzymatic specificity in the cys-met-metabolism PLP-dependent enzymes family: crystal structure of cystathionine γ-lyase from yeast and intrafamiliar structure comparison. Biol Chem.

[42] Molina DM, Jafari R, Ignatushchenko M, Seki T (2013). Monitoring drug target engagement in cells and tissues using the cellular thermal shift assay. Science.

[43] Moreno JC, Rojas BE, Vicente R, Gorka M, Matz T, Chodasiewicz M, Peralta-Ariza JS, Zhang Y, Alseekh S, Childs D, Luzarowski M, Nikoloski Z, Zarivach R, Walther D, Hartman MD, Figueroa CM, Iglesias AA, Fernie AR, Skirycz A (2021). Tyr-Asp inhibition of glyceraldehyde 3-phosphate dehydrogenase affects plant redox metabolism. EMBO J.

[44] Murphy JP, Stepanova E, Everley RA, Paulo JA, Gygi SP (2015). Comprehensive temporal protein dynamics during the diauxic shift in *Saccharomyces cerevisiae*. Mol Cell Proteomics.

[45] Naka K, Jomen Y, Ishihara K, Kim J, Ishimoto T, Bae EJ, Mohney RP, Stirdivant SM, Oshima H, Oshima M, Kim DW, Nakauchi H, Takihara Y, Kato Y, Ooshima A, Kim SJ (2015). Dipeptide species regulate P38MAPK-Smad3 signalling to maintain chronic myelogenous leukaemia stem cells. Nat Commun.

[46] O’Connell JD, Tsechansky M, Royall A, Boutz DR, Ellington AD, Marcotte EM (2014). A proteomic survey of widespread protein aggregation in yeast. Mol BioSyst.

[47] Ono B-I-I, Naito K, Shirahige Y-I-I, Yamamoto M (1991). Regulation of cystathionine Γ-lyase in *Saccharomyces cerevisiae*. Yeast.

[48] Ono B-I, Tanaka K, Naito K, Heike C, Shinoda S, Yamamoto S, Ohmiro S, Oshima T, Toh-E A (1996). Cloning and characterization of the ALG3 gene of *Saccharomyces cerevisiae*. Glycobiology.

[49] Potel CM, Kurzawa N, Becher I, Typas A, Mateus A, Savitski MM (2021). Impact of phosphorylation on thermal stability of proteins. Nat Methods.

[50] Rappsilber J, Ishihama Y, Mann M (2003). Stop and go extraction tips for matrix-assisted laser desorption/ionization, nanoelectrospray, and LC/MS sample pretreatment in proteomics. Anal Chem.

[51] Sahu I, Mali SM, Sulkshane P, Xu C, Rozenberg A, Morag R, Sahoo MP, Singh SK, Ding Z, Wang Y, Day S, Cong Y, Kleifeld O, Brik A, Glickman MH (2021). The 20S as a stand-alone proteasome in cells can degrade the ubiquitin tag. Nat Commun.

[52] Salas D, Greg Stacey R, Akinlaja M, Foster LJ (2020). Next-generation interactomics: considerations for the use of co-elution to measure protein interaction networks. Mol Cell Proteomics.

[53] Sampaio-Marques B, Ludovico P (2018). Linking cellular proteostasis to yeast longevity. FEMS Yeast Res.

[54] Savitski MM, Reinhard FBM, Franken H, Werner T, Savitski MF, Eberhard D, Molina DM, Jafari R, Dovega RB, Klaeger S, Kuster B, Nordlund P, Bantscheff M, Drewes G (2014). Tracking cancer drugs in living cells by thermal profiling of the proteome. Science.

[55] Schlossarek D, Luzarowski M, Sokołowska E, Górka M, Willmitzer L, Skirycz A (2021). PROMISed: a novel web-based tool to facilitate analysis and visualization of the molecular interaction networks from co-fractionation mass spectrometry (CF-MS) experiments. Comput Struct Biotechnol J.

[56] Smith IR, Hess KN, Bakhtina AA, Valente AS, Rodríguez-Mias RA, Villén J (2021). Erratum to: High Throughput Discovery of Functional Protein Modifications by Hotspot Thermal Profiling (Nature Methods, (2019), 16, 9, (894–901), DOI: 10.1038/S41592-019-0499-3). Nat Methods.

[57] Sokolowska EM, Schlossarek D, Luzarowski M, Skirycz A (2019). PROMIS: global analysis of PROtein-metabolite interactions. Curr Protocols Plant Biol.

[58] Strehmel N, Hoehenwarter W, Mönchgesang S, Majovsky P, Krüger S, Scheel D, Lee J (2017). Stress-related mitogen-activated protein kinases stimulate the accumulation of small molecules and proteins in *Arabidopsis thaliana* root exudates. Front Plant Sci.

[59] Sun W, Dai L, Han Yu, Puspita B, Zhao T, Li F, Tan JL, Lim YT, Chen MW, Sobota RM, Tenen DG, Prabhu N, Nordlund P (2019). Monitoring structural modulation of redox-sensitive proteins in cells with MS-CETSA. Redox Biol.

[60] Thirumalaikumar VP, Wagner M, Balazadeh S, Skirycz A (2021). Autophagy is responsible for the accumulation of proteogenic dipeptides in response to heat stress in *Arabidopsis thaliana*. FEBS J.

[61] Toney MD (2005). Reaction specificity in pyridoxal phosphate enzymes. Arch Biochem Biophys.

[62] Toney MD (2011). Controlling reaction specificity in pyridoxal phosphate enzymes. Biochimica et Biophysica Acta - Proteins and Proteomics.

[63] Verma K, Saxena K, Donaka R, Chaphalkar A, Rai MK, Shukla A, Zaidi Z, Dandage R, Shanmugam D, Chakraborty K (2020). Distinct metabolic states of a cell guide alternate fates of mutational buffering through altered proteostasis. Nat Commun.

[64] Veyel D, Kierszniowska S, Kosmacz M, Sokolowska EM, Michaelis A, Luzarowski M, Szlachetko J, Willmitzer L, Skirycz A (2017). System-wide detection of protein-small molecule complexes suggests extensive metabolite regulation in plants. Sci Rep.

[65] Veyel D, Sokolowska EM, Moreno JC, Kierszniowska S, Cichon J, Wojciechowska I, Luzarowski M, Kosmacz M, Szlachetko J, Gorka M, Méret M, Graf A, Meyer EH, Willmitzer L, Skirycz A (2018). PROMIS, global analysis of PROtein-metabolite interactions using size separation in *Arabidopsis thaliana*. J Biol Chem.

[66] Volkening JD, Stecker KE, Sussman MR (2019). Proteome-wide analysis of protein thermal stability in the model higher plant *Arabidopsis thaliana*. Mol Cell Proteomics.

[67] Wagner M, Zhang B, Tauffenberger A, Schroeder FC, Skirycz A (2021). experimental methods for dissecting the terraincognita of protein-metabolite interactomes. Curr Opin Syst Biol.

[68] Wan C, Borgeson B, Phanse S, Fan Tu, Drew K, Clark G, Xiong X, Kagan O, Kwan J, Bezginov A, Chessman K, Pal S, Cromar G, Papoulas O, Ni Z, Boutz DR, Stoilova S, Havugimana PC, Guo X, Malty RH, Sarov M, Greenblatt J, Mohan Babu W, Derry B, Tillier ER, Wallingford JB, Parkinson J, Marcotte EM, Emili A (2015). Panorama of ancient metazoan macromolecular complexes. Nature.

[69] Ward JH (1963). Hierarchical grouping to optimize an objective function. J Am Stat Assoc.

[70] Werner-Wahsburne M, Braun E, Johnston GC, Singer RA (1996). Stationary phase in the yeast *Saccharomyces cerevisiae*. Microbiol Rev.

[71] Williams A, Chiles EN, Conetta D, Pathmanathan JS, Cleves PA, Putnam HM, Xiaoyang S, Bhattacharya D (2021). Metabolomic shifts associated with heat stress in coral holobionts. Sci Adv.

[72] Xia Z, Webster A, Fangyong Du, Piatkov K, Ghislain M, Varshavsky A (2008). Substrate-binding sites of UBR1, the ubiquitin ligase of the N-end rule pathway. J Biol Chem.

[73] Yang L, Fountain JC, Ji P, Ni X, Chen S, Lee RD, Kemerait RC, Guo B (2018). Deciphering drought-induced metabolic responses and regulation in developing Maize Kernels. Plant Biotechnol J.

[74] Yu G, Wang LG, Han Y, He QY (2012). ClusterProfiler: an R package for comparing biological themes among gene clusters. OMICS.

[75] Zampar GG, Kümmel A, Ewald J, Jol S, Niebel B, Picotti P, Aebersold R, Sauer U, Zamboni N, Heinemann M (2013). Temporal system-level organization of the switch from glycolytic to gluconeogenic operation in yeast. Mol Syst Biol.

