# Supplementary figures and images for: Rewiring of the protein–protein–metabolite interactome during the diauxic shift in yeast
Source: Cell Mol Life Sci. 2022 Oct 15;79(11):550. doi: 10.1007/s00018-022-04569-8 (PMC9569316; doi:10.1007/s00018-022-04569-8)

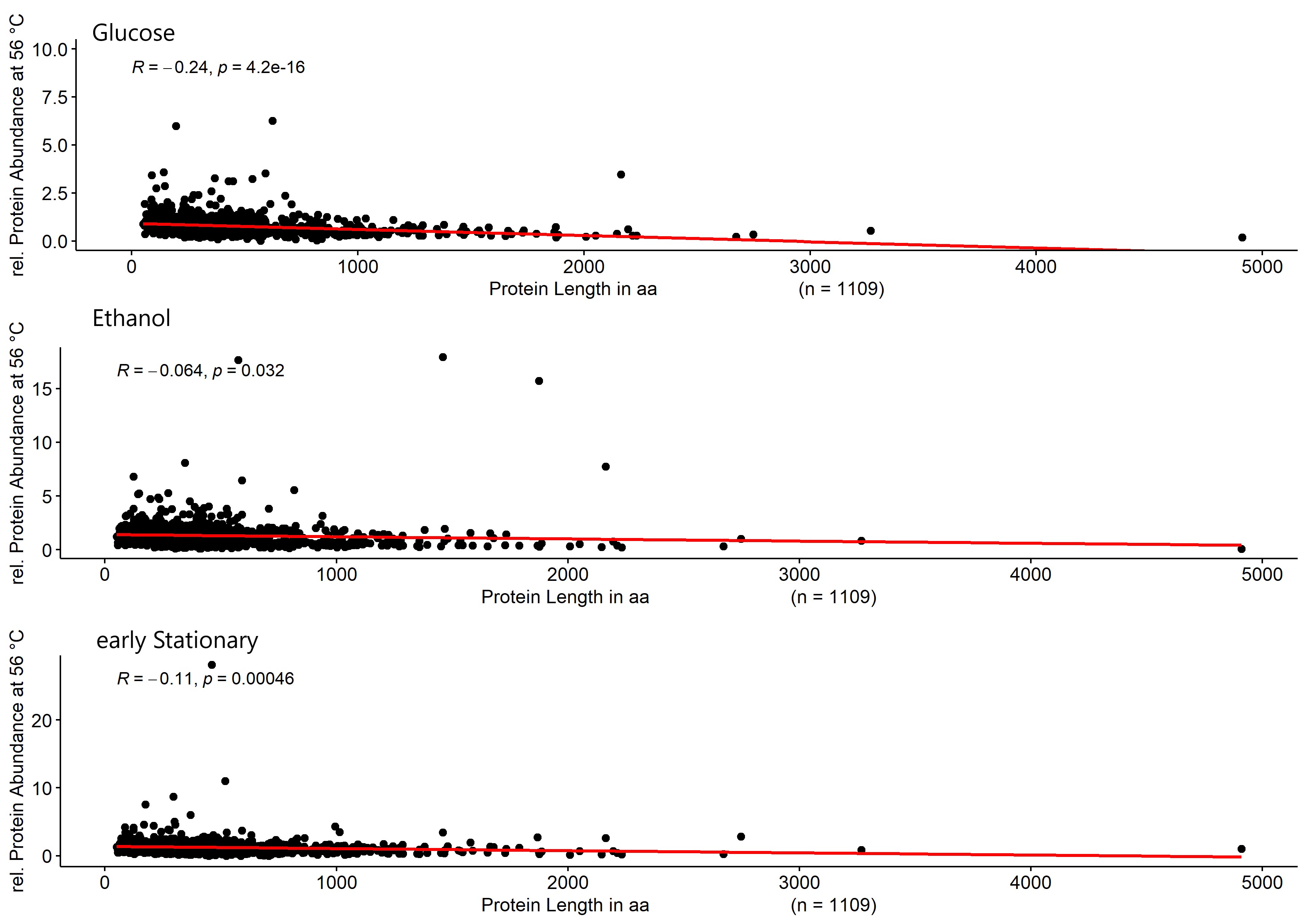

Supplement: Supplementary file 2 — Supplementary file2 Supplementary Figure 2: Protein thermal stability and protein length show a weak, but statistically significant negative correlation. Scatter plots showing the relative protein abundance after treatment at 56 °C versus protein length in amino acids. For the early stationary phase, the protein MAK21 was excluded from the analysis. (JPG 496 KB) [file 18_2022_4569_MOESM2_ESM.jpg]

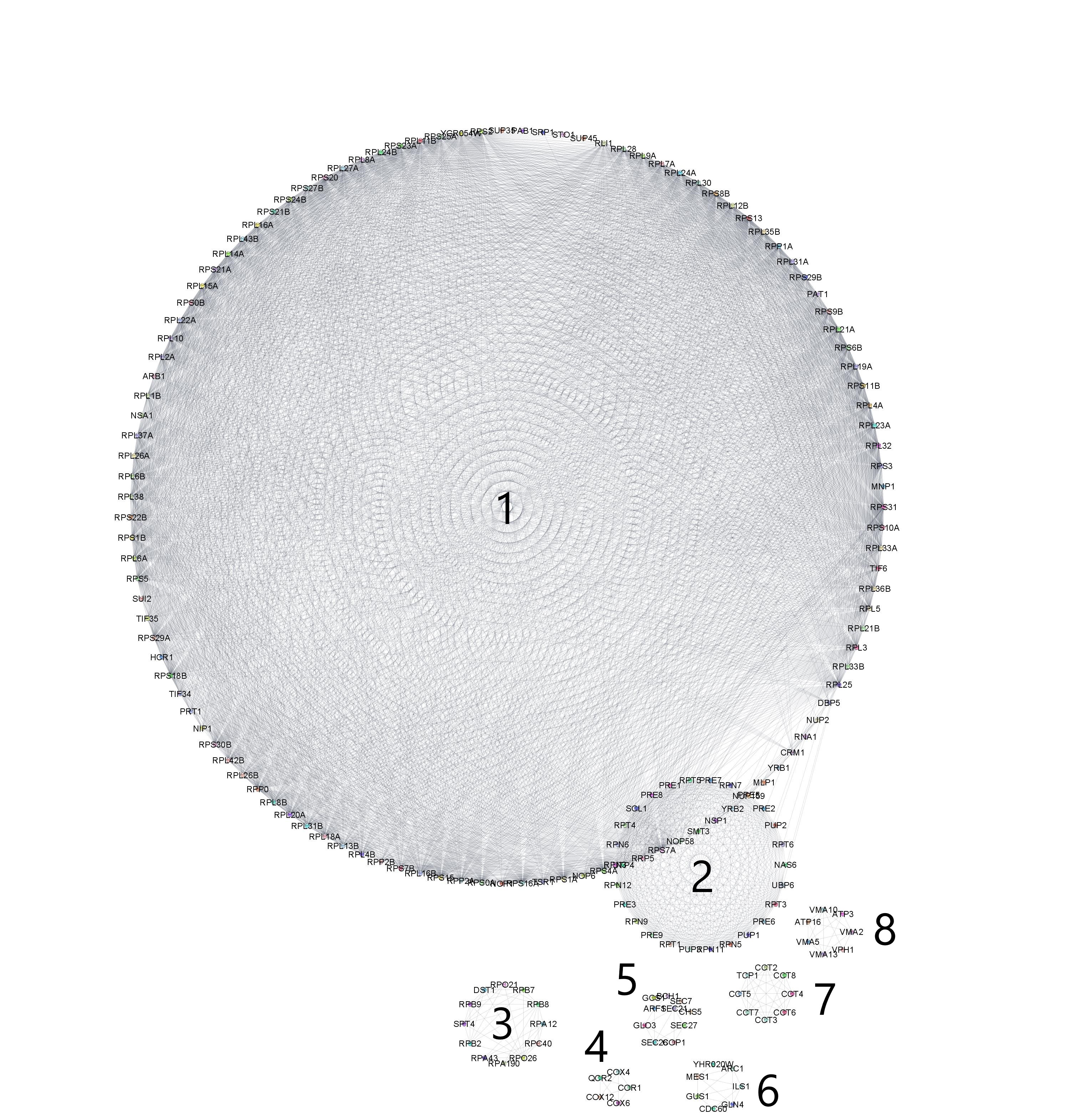

Supplement: Supplementary file 3 — Supplementary file3 Supplementary Figure 3: Subunits of the macromolecular complexes identified as differential between early stationary and glucose phases in the iTSA experiment. PPIs (experimental evidence, confidence score >0.9) were downloaded from STRING database. Figure was generated using Cytoscape. 1 Ribosome, 2 Proteasome, 3 RNA polymerase complex, 4 Aminoacyl-tRNA synthetase complex, 5 Cytochrome c reductase complex, 6 Coatomer, 7 CTT chaperone complex, 8 V-ATPase (JPEG 2065 KB) [file 18_2022_4569_MOESM3_ESM.jpeg]

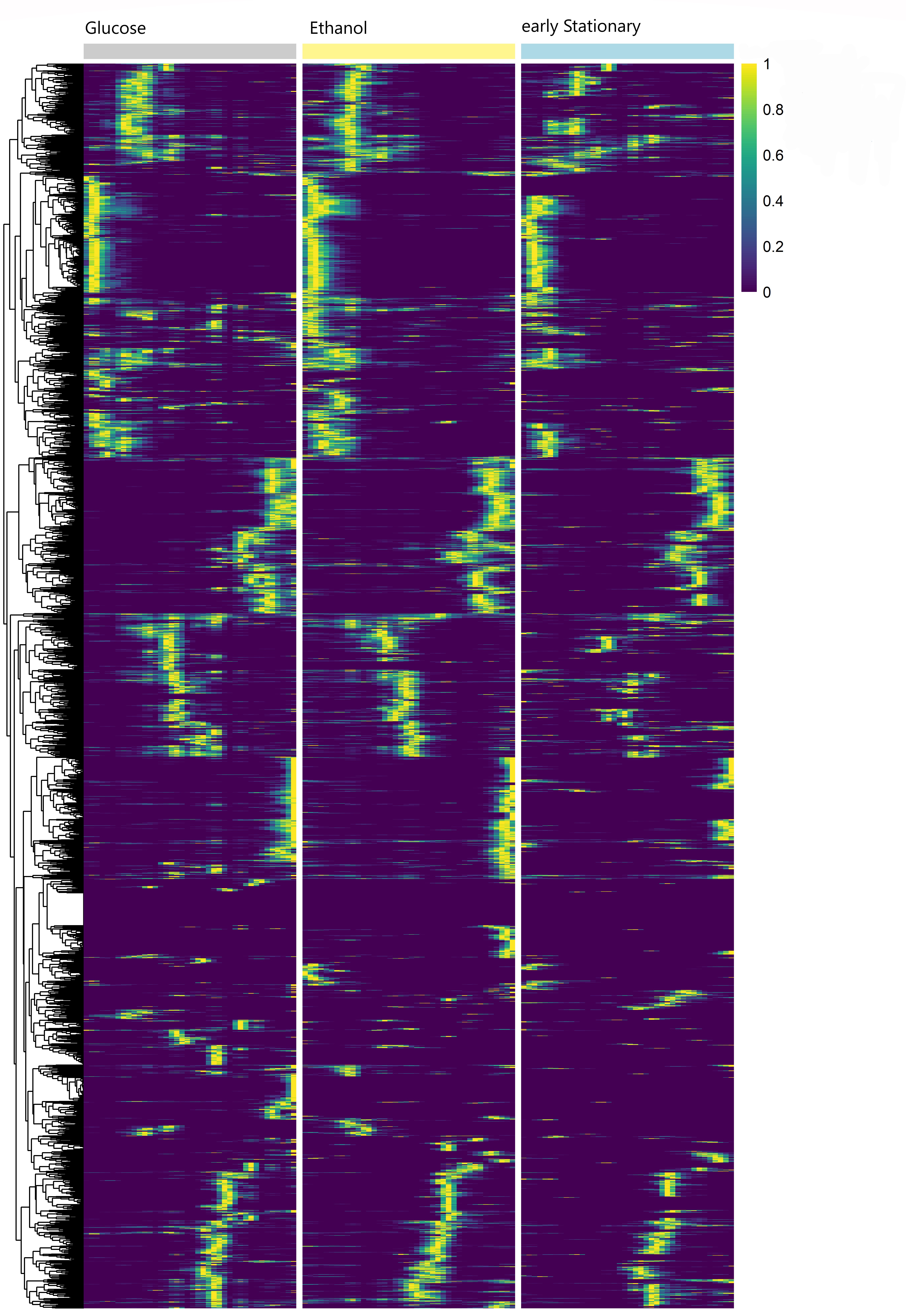

Supplement: Supplementary file 4 — Supplementary file4 Supplementary Figure 4: Clustered heatmap of normalized protein fractionation patterns. Protein fractionation patterns of the three growth phases (grey, glucose; yellow, ethanol; blue, early stationary) are concatenated and clustered using euclidean distance and the “complete” hierarchical clustering method as implemented in the pheatmap R-pacakge. (JPG 3816 KB) [file 18_2022_4569_MOESM4_ESM.jpg]

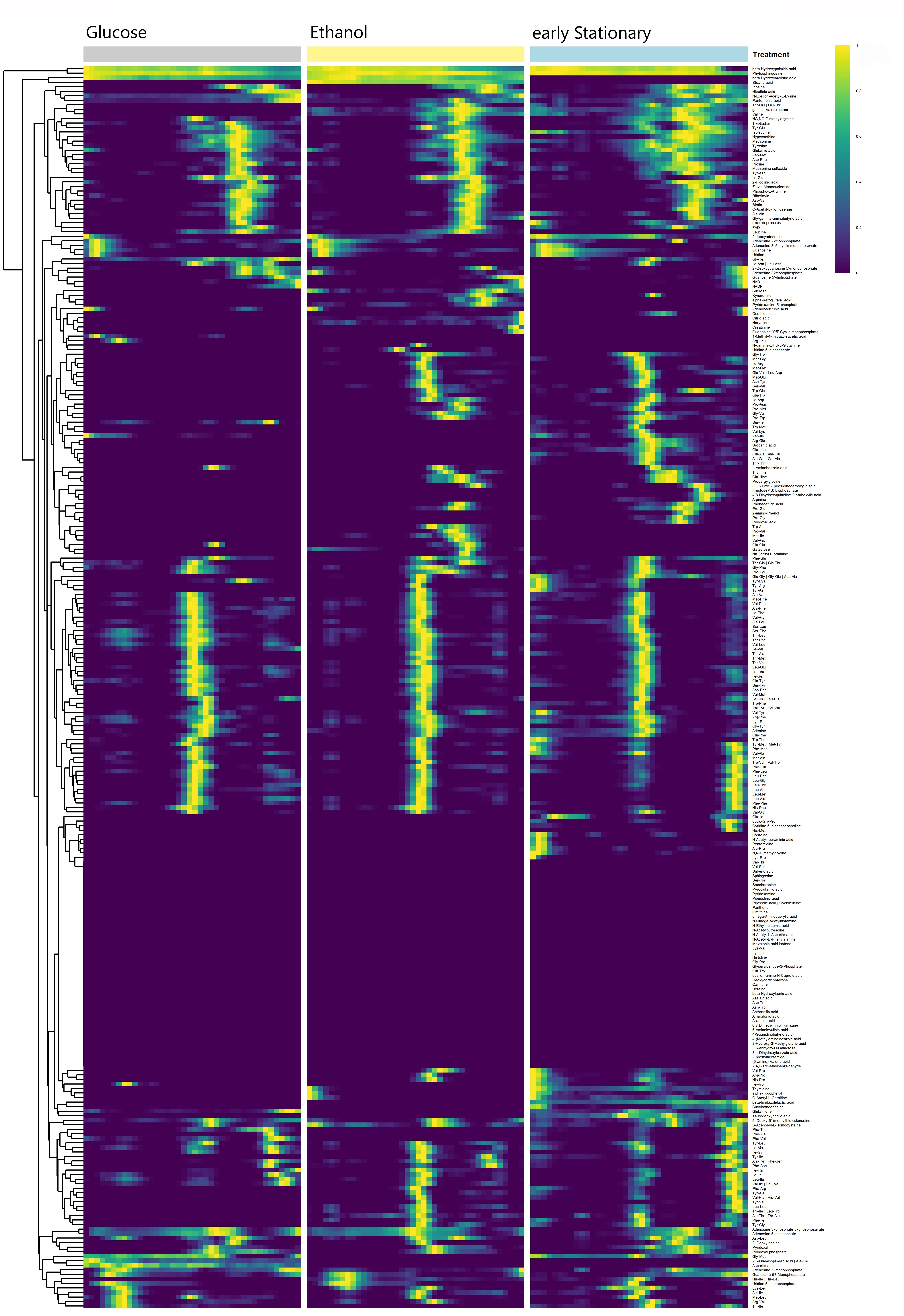

Supplement: Supplementary file 5 — Supplementary file5 Supplementary Figure 5: Clustered heatmap of normalized metabolite fractionation patterns in the protein containing fractions. Metabolite fractionation patterns of the three growth phases (grey, glucose; yellow, ethanol; blue, early stationary) are concatenated and clustered using euclidean distance and the “complete” hierarchical clustering method as implemented in the pheatmap R-pacakge. Note that metabolites which are depicted as absent in the heatmap are present in the non-protein containing fractions, or the fractionation profiles between replicates may not be reproducible. (JPG 2584 KB) [file 18_2022_4569_MOESM5_ESM.jpg]

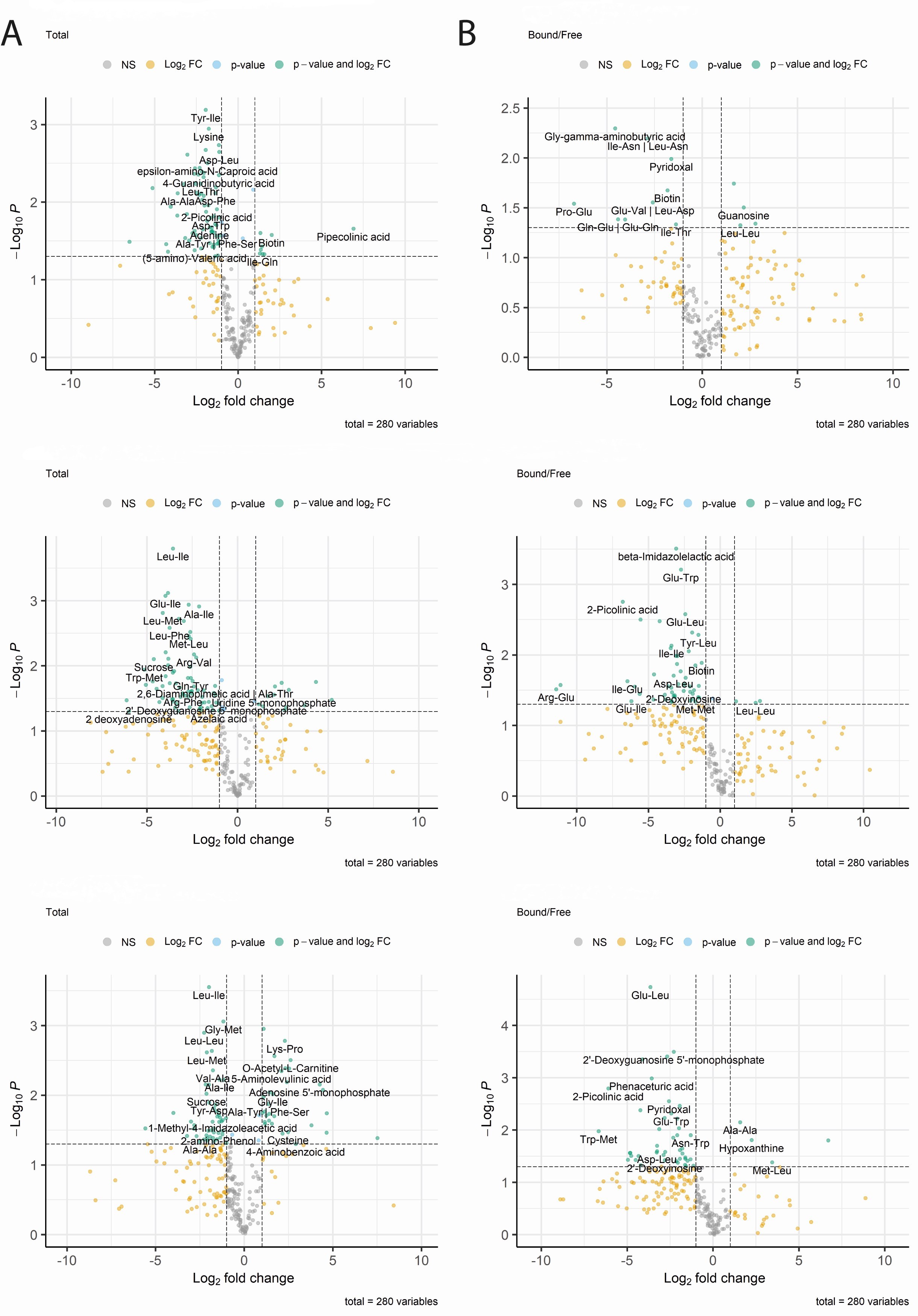

Supplement: Supplementary file 6 — Supplementary file6 Supplementary Figure 6: A) Volcano plot showing the differences in total metabolite abundance between glucose and ethanol phase (top), glucose and early stationary phase (middle), and ethanol and early stationary phase (bottom), respectively. Total abundances were estimated as the sum of the metabolite fractionation profile. B) Changes in metabolite interaction status between glucose and ethanol phase (top), glucose and early stationary phase (middle), and ethanol and early stationary phase (bottom), respectively, as measured as the ratio between metabolite abundances in protein bound and protein free fractions. (JPG 842 KB) [file 18_2022_4569_MOESM6_ESM.jpg]

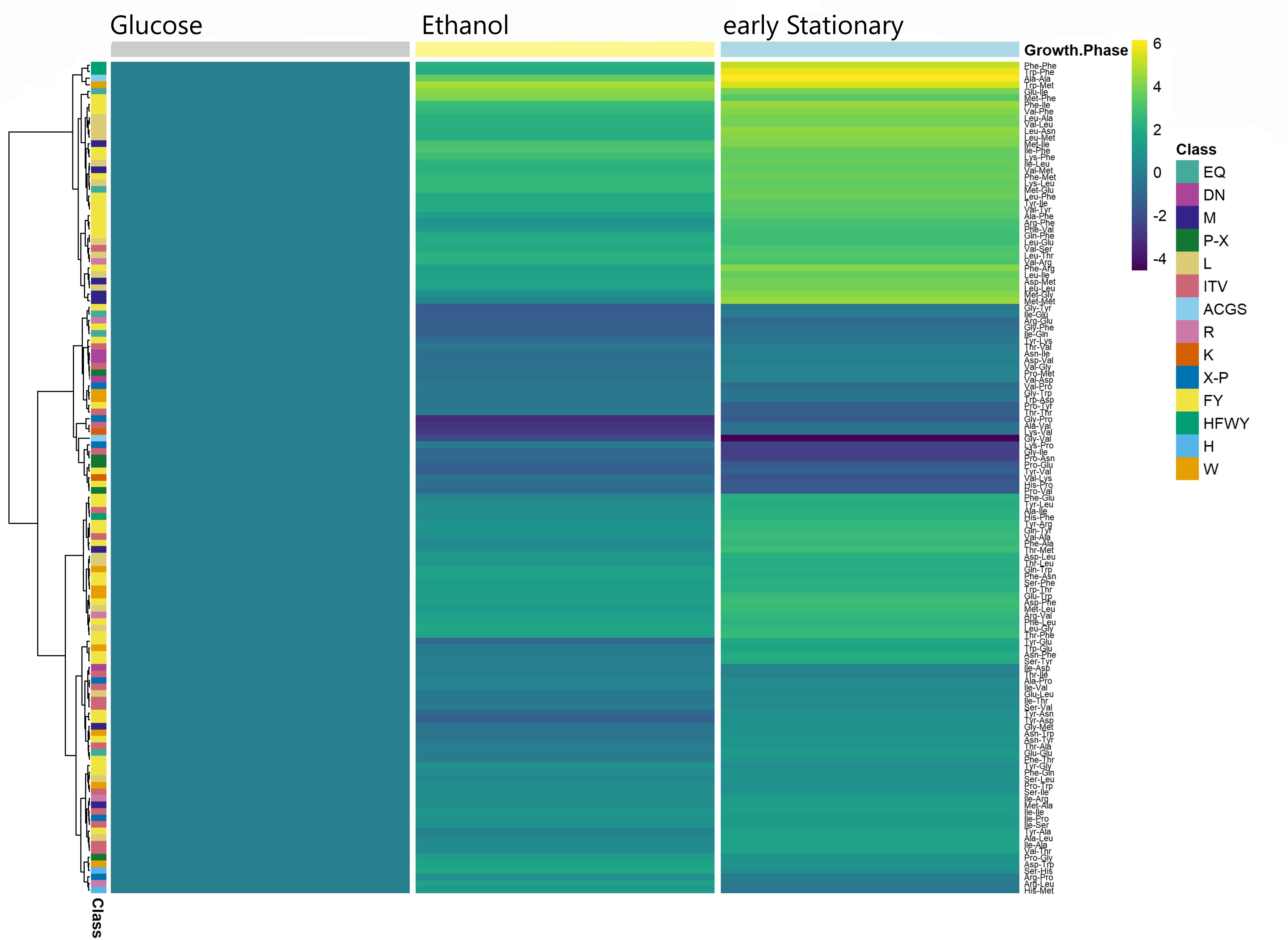

Supplement: Supplementary file 7 — Supplementary file7 Supplementary Figure 7: Heat-map of dipeptide accumulatio across the yeast growth stages. Data are expressed as log2 fold change in comparison to the glucose-utilizing phase. (JPG 839 KB) [file 18_2022_4569_MOESM7_ESM.jpg]

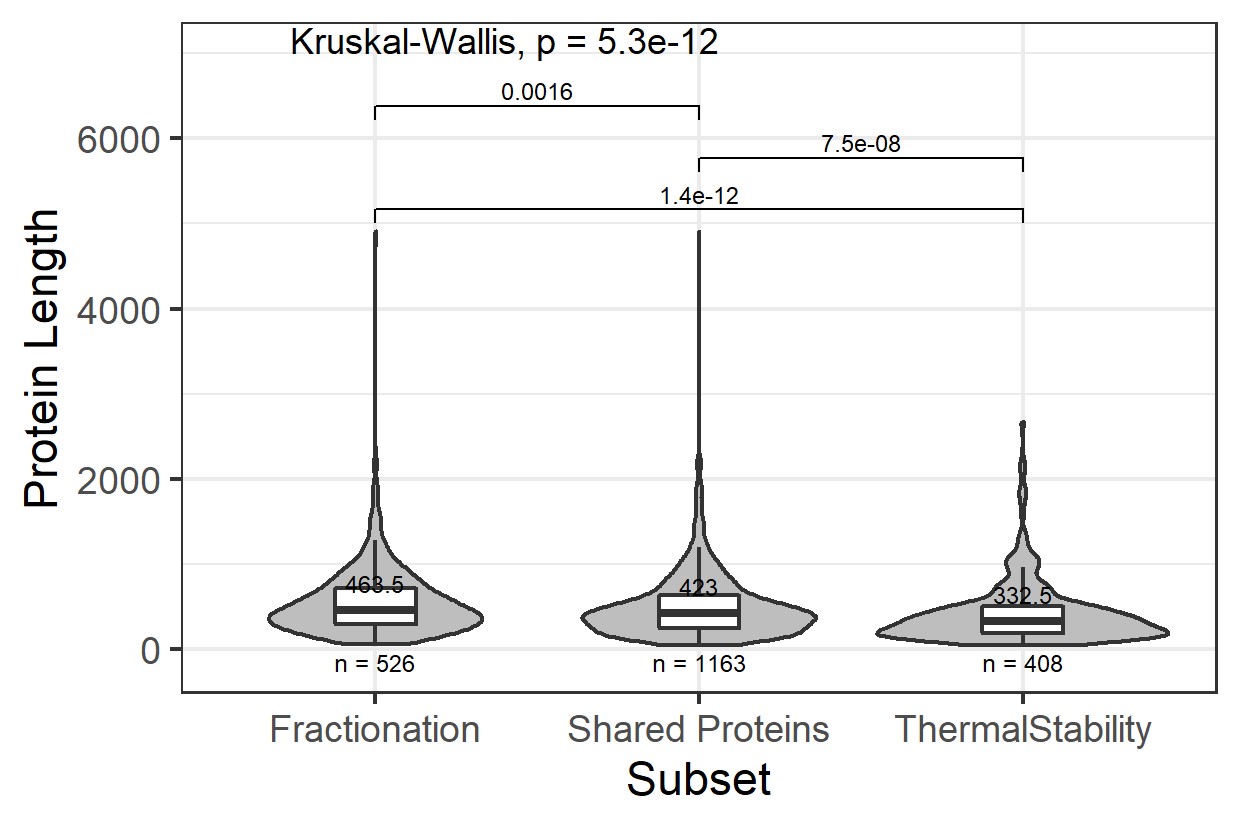

Supplement: Supplementary file 8 — Supplementary file8 Supplementary Figure 8: Violin plot showing protein length in amino acids for proteins with differential fractionation only (Fractionation), differences in thermal stability only (ThermalStability), and proteins affected by either or both experiments (Shared Proteins) between the glucose and late ethanol -utilizing phase (see also Figure 4 A and Table S13). Proteins affected by fractionation only are significantly larger than proteins affected by thermal stability only. (JPG 109 KB) [file 18_2022_4569_MOESM8_ESM.jpg]
